# Secreted nuclease effector neutralization by active site mimicry in Bacillota

**DOI:** 10.1128/mbio.01516-26

**Published:** 2026-08-17

**Authors:** Romina Abbasian, Bishal Parajuli, Liping Yu, Brice Durocher, Emma Gardner, Ethan Chodur, Maddisen K. Dwelley, Craig D. Ellermeier, Theresa D. Ho, Dustin E. Bosch

**Affiliations:** 1 Pathology Department, University of Iowa 160416, Iowa City, Iowa, USA; 2 Department of Microbiology and Immunology, University of California 214560, San Francisco, California, USA; 3 Biochemistry and Molecular Biology Department, University of Iowa 196208, Iowa City, Iowa, USA; 4 Molecular Physiology and Biophysics Department, University of Iowa 174490, Iowa City, Iowa, USA; 5 NMR Core Facility, Carver College of Medicine, University of Iowa 4083, Iowa City, Iowa, USA; 6 Holden Comprehensive Cancer Center 502602, Iowa City, Iowa, USA; 7 Microbiology and Immunology Department, University of Iowa 311821, Iowa City, Iowa, USA; Michigan State University, East Lansing, Michigan, USA

**Keywords:** polymorphic toxin, nuclease, mimicry, interbacterial antagonism

## Abstract

Polymorphic toxins mediate interbacterial antagonism among competitors in the gut microbiome. Nuclease effectors, distantly related to the type VI-secreted Bacteroidales Tde, are enriched in human gut Bacillota. Tde mediates antagonism among Bacillota, and expression of the cognate immunity, Tdi, in recipients is protective. Crystal structures of Tde/Tdi complexes from two *Bacillus* spp. and *Enterococcus quebecensis* highlight a conserved mechanism of immunity. Tdi engages Tde with high-affinity, specific binding at an interface that features predominantly polar amino acids. A separate Tdi interface has a very highly conserved P(Φ)_4_GG motif that structurally mimics and displaces a short helix in Tde’s active site, which contains the critical catalytic residues. An isolated P(Φ)_4_GG motif peptide is sufficient for Tde nuclease activity inhibition at high concentrations. However, key residues at both the polar interface and P(Φ)_4_GG are required for complete inhibition of nuclease activity and protection against toxicity. We propose a multivalent Tde/Tdi neutralization mechanism where an initial high-affinity interface increases the local concentration of Tdi’s P(Φ)_4_GG motif, enabling it to displace the Tde active site through structural mimicry. The resulting conformational rearrangement of Tde increases its flexibility in solution and susceptibility to proteolysis, which may aid in eliminating the toxic effector.

**IMPORTANCE:**

Bacteria in the gut microbiome compete using toxin secretion systems. Prior research has emphasized the importance of secretion systems in gram-negative bacteria. We describe a class of secreted nuclease effectors (toxins) and protective immunity proteins that are enriched in gram-positive Bacillota in human gut microbiomes. These effector/immunity pairs mediate antagonism among *Bacillus* and *Enterococcus* spp. The immunity proteins neutralize the nuclease effector through a unique mechanism of enzymatic active site mimicry. The immunity proteins bind effectors with very high-affinity at an interface with polar residues. The effector undergoes a large conformational change. A very highly conserved motif on the immunity surface competitively displaces an active site short helix and loop that contains the key catalytic residues. This rearrangement of the effector renders it inactive and susceptible to elimination by proteolysis.

## INTRODUCTION

Bacteria in polymicrobial ecosystems, such as the intestinal microbiome, compete for resources. Direct killing of competing bacteria can occur by the delivery of polymorphic toxins (effectors) from the donor that disrupt essential cellular processes, such as cell division and peptidoglycan homeostasis, in a recipient bacterium (1). Donor intoxication of self and kin is prevented by neutralizing immunity proteins. The evolutionary success of the polymorphic toxin delivery strategy is reflected by highly diverse effector and immunity families and elaborate toxin secretion system (TSS) molecular machinery (1, 2). Families of related effectors are often exported by varied secretion systems in bacteria from different phyla (2). Secreted effectors are usually enzymes and exhibit a striking range of structures and functions (1, 2). Polymorphic effector/immunity structural and biochemical studies to date have converged on a primary mechanism of effector neutralization. Most immunity proteins bind directly at or immediately adjacent to effector active sites and sterically prevent access to substrates (1, 3–6). Exceptions to this rule include enzymatic reversal of effector activity by immunity (7, 8), several examples of immunity proteins inducing structural rearrangement of effectors to disrupt the active site (3, 9, 10), and allosteric inhibition or competitive exclusion of substrate binding via a binding site away from the effector active site (11, 12). Effector/immunity interactions typically have very high affinity and thermal stability (3), reflecting the necessity of efficiently sequestering highly cytotoxic effectors within the cell.

Bacteroidales frequently encode type VI secretion systems (T6SS), dedicated to interbacterial antagonism (13, 14). Prior metagenomic studies have demonstrated differential abundance of T6SS and effector/immunity genes in the dysbiosis of multiple human diseases, such as inflammatory bowel disease and type 2 diabetes (15). Human gut metagenome associative analyses, culture-based competitive growth experiments, and gnotobiotic animal models indicate that Bacteroidales T6SS, effectors, and immunity genes are all critical determinants of colonization and strain abundance in the intestine (16–19). Strains encoding dominant effector types competitively exclude colonization by related but non-immune Bacteroidales (14, 17, 18, 20), potentially contributing to individual microbiome stability. Our previous study characterized a T6SS DNase effector (Tde) in Bacteroidales that mediates antagonism among related species (3). T6SS DNase immunity proteins (Tdi) bind and neutralize Tde. Structures of two Tde·Tdi complexes and Tde only from Bacteroidales demonstrated structural rearrangement of the DNase effector and disruption of the DNA-binding and active sites upon interaction with immunity (3). Tdi inserts into the central core of Tde, splitting two subdomains and converting the active site into a flexible loop. However, the detailed structural mechanisms underlying this mode of effector neutralization were unknown.

Distantly related Tde/Tdi pairs are also found in gram-positive Bacillota, but their functions and secretion mechanisms are unknown, particularly in the context of the gut microbiome. We hypothesized that Bacillota Tde/Tdi mediate interbacterial antagonism in the gut microbiome with conserved effector and immunity mechanisms. To test the hypothesis, we undertook a structure/function study of Tde·Tdi in gut Bacillota.

## RESULTS

### Bacillota and Bacteroidales MAGs encoding Tde/Tdi are enriched for different secretion systems

**Fig 1:**
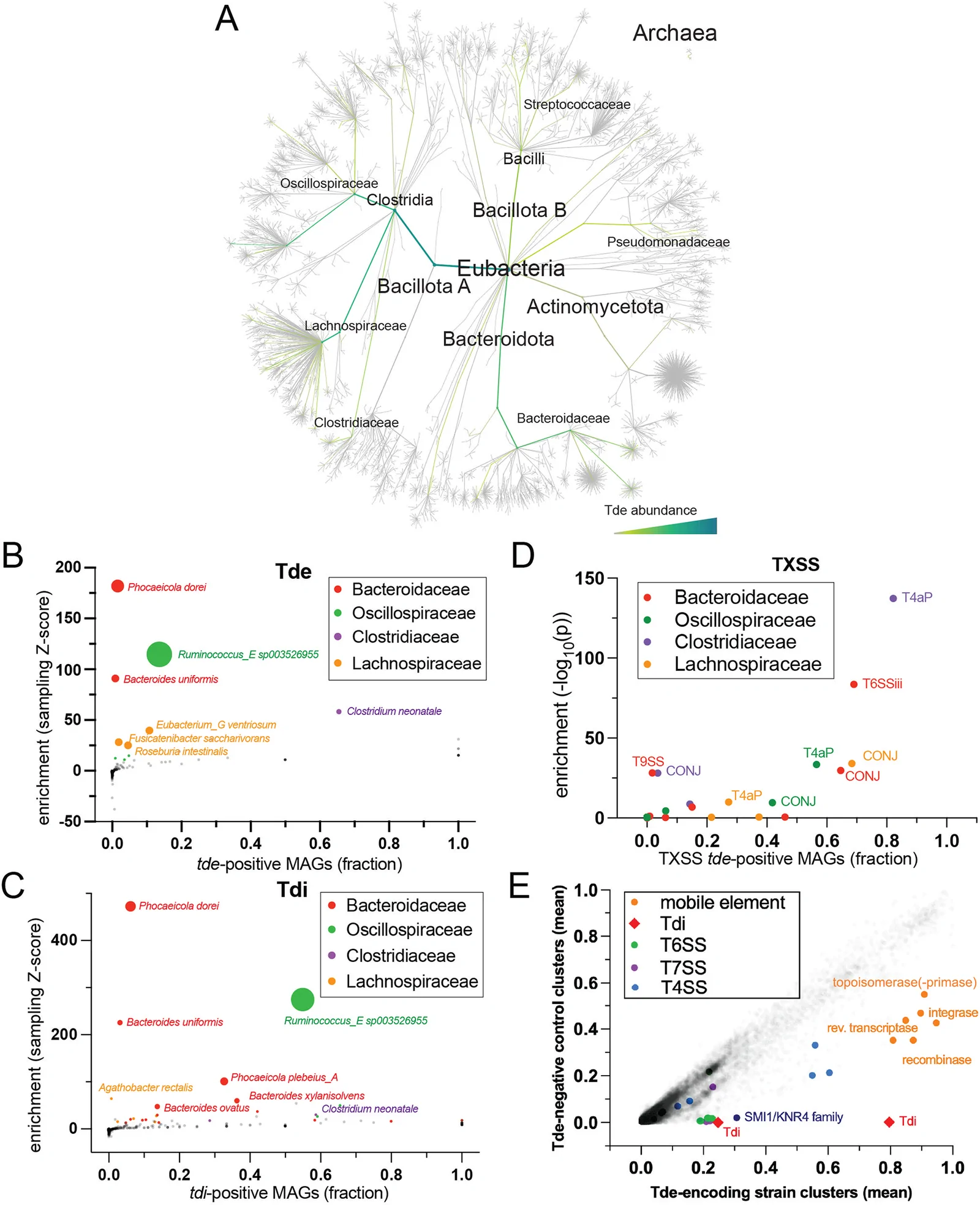
Gut MAGs that encode tde are enriched for T6SS, T7SS, T4SS, and mobile element-related genes. (**A**) Tde homologs identified in 200,000 metagenome-assembled genomes (MAGs) are distributed among Bacteroidota and Bacillota phyla. (**B and C**) *tde* and *tdi* are enriched in four families compared to the positivity rate for all MAGs. Dot size is scaled to the number of positive MAGs. (**D**) TXSSscan applied to *tde*-positive MAGs and *tde*-negative controls matched for genus revealed enrichment of T6SS in tde-positive Bacteroidaceae and T4SS (CONJ = conjugative type IV, T4aP = type IVa pilus) in all four families. T9SS is also enriched and restricted to certain Bacteroidota (*Porphyromonas*, *Flavobacterium*). (**E**) All coding sequences from *tde*-positive MAGs and controls were clustered with mmseqs2. Scatter toward the lower right corner reflects enrichment in tde-positive strains. Two clusters mapping to Tdi sequences are highly associated with Tde. T4SS-, T6SS-, and T7SS-specific clusters also associate with *tde*. Mobile element-related sequences are enriched, consistent with manual inspection of selected MAGs.

Although Tde/Tdi have been studied to date only in gram-negative bacteria with T6SS, we anecdotally observed homologs in some Bacillota in a collection of ~1,200 human intestinal commensal genomes (3). We hypothesized that certain gut Bacillota taxa may encode Tde/Tdi that mediate antagonism. To identify bacteria in gut microbiomes that carry *tde/tdi*, we applied local sequence alignment and hidden Markov model searches (3, 15) to ~200,000 metagenome-assembled genomes (MAGs) in the MGnify database (21). A gene for *tde* was identified in 1,010 MAGs, with the vast majority encoding adjacent *tdi* (Fig. 1A). There was enrichment of *tde* in the Bacteroidaceae and three Bacillota families: Oscillospiraceae, Lachnospiraceae, and Clostridiaceae (Fig. 1B). Genes for *tdi* had a similar taxonomic distribution, but orphan *tdi* were 10-fold more abundant than *tde/tdi* pairs (~10,000 MAGs; Fig. 1C). MAG binning methods are biased against the inclusion of mobile elements (22, 23), so the prevalence of *tdei/tdi* among gut bacteria may be underestimated in this analysis.

Bacteroidaceae are known to secrete Tde with T6SS, but Bacillota lack this system. We hypothesized that Bacillota may use a different secretion system for Tde secretion, which would be enriched in Tde-positive MAGs. To systematically assess secretion systems, we applied TXSScan (24) to all *tde-*positive MAGs and a 1:1 genus-matched random selection of control MAGs. As expected, the Bacteroidales-restricted T6SS^iii^ was associated with *tde* (Fig. 1D). All four dominant *tde*-encoding families showed enrichment for T4SS in *tde-*positive MAGs, including conjugative types (CONJ) and type IVa pilus (T4aP), a multifunctional system used for motility, attachment, DNA acquisition, and protein secretion (25). To detect potential non-canonical TSS proteins associated with Tde, we clustered all coding sequences from *tde*-positive and control MAGs using mmseqs2 (26) and compared their abundances by *tde* status (Fig. 1E). As a positive control, two clusters mapping to Tdi were highly associated with Tde. A SMI1/KNR4 family cluster was enriched, consistent with prior research showing genomic proximity of this gene family to polymorphic toxins (27). T4SS-, T6SS-, and T7SS-related sequence clusters and mobile genetic element-related genes were also enriched in *tde*-positive MAGs.

In summary, *tde/tdi* are predominantly found in Bacteroidaceae and three families of Bacillota in the gut microbiome. Some Bacillota Tde co-occur with T7SS, but most do not. This weak association suggests that T7SS may be a secretion mechanism in some Bacillota, but other mechanisms likely exist. The enrichment analysis shows an association between *tde/tdi* and T4SS, suggesting possible involvement in secretion or horizontal gene transfer. Outside of the gut microbiome, *tde/tdi* are more widely distributed among >10 families of Bacillota (Fig. S1). Effector/immunity sequence similarity aligns poorly with bacterial taxonomy, suggesting that horizontal transfer of *tde/tdi* is also a frequent occurrence in other environments.

### Tde/Tdi mediate interbacterial antagonism among Bacillota

**Fig 2:**
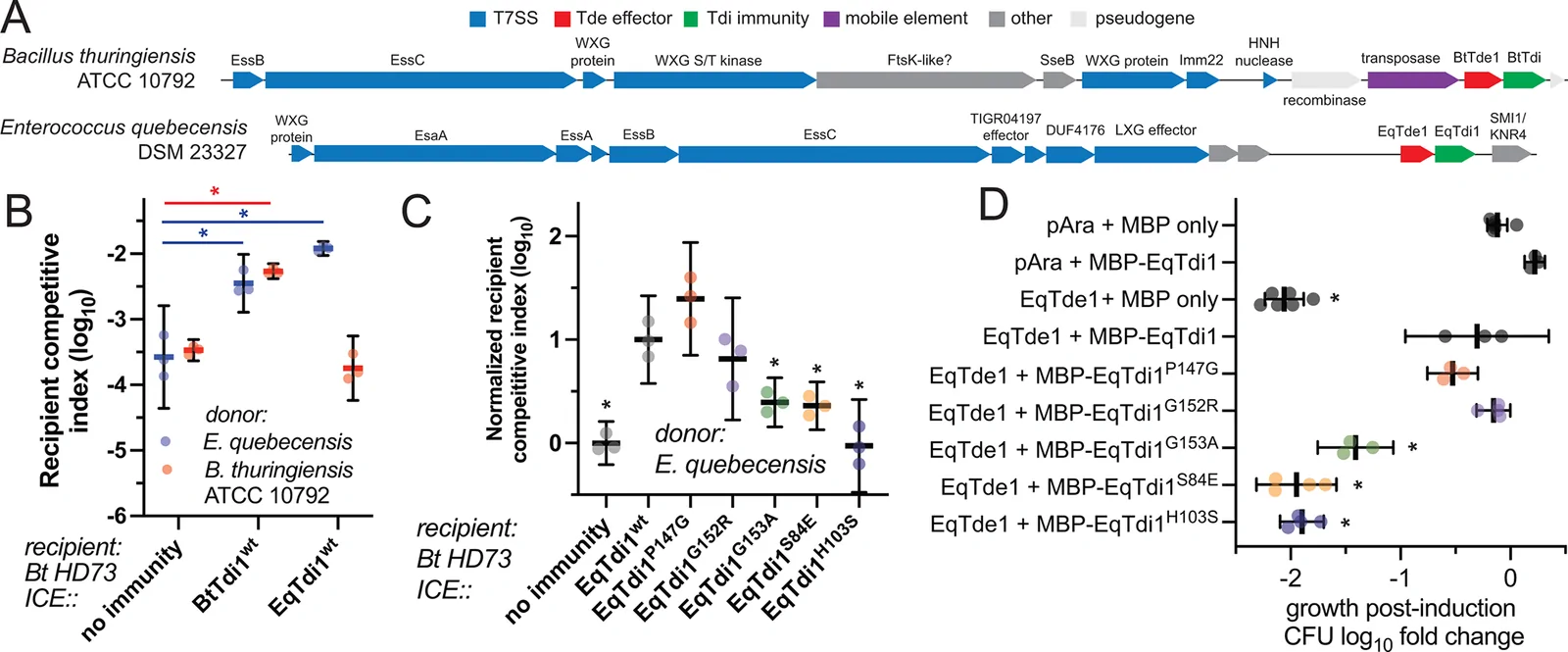
Bacillota Tdi protects against Tde-mediated toxicity and interbacterial antagonism. (**A**) Tde/Tdi pairs are encoded adjacent to type VII secretion systems in *E. quebecensis* and *B. thuringiensis* ATCC 10792. (**B**) Expression of the cognate immunity BtTdi1 from an ICE element in the recipient Bt HD73 strain provides an advantage in competitive growth experiments with either Bt ATCC 10792 or *E. quebecensis.* Antagonism by *E. quebecensis*, but not Bt ATCC 10792, against Bt HD73 was impaired by expression of EqTdi1. (**C**) Polar interface residues of EqTdi1 S84 and H103, but not components of the EqTdi1 P(Φ)_4_GG motif, were required for full protection by EqTdi1. (**D**) EqTde1 overexpressed in *E. coli* using a “no-leak” arabinose-inducible promoter was cytotoxic. EqTde1 toxicity was reversed by co-expression of an MBP-EqTdi1 fusion. Mutation of G153, but not P147 in the EqTdi P(Φ)_4_GG motif, reduced protection against EqTde1. * *q* < 0.05 compared to EqTdi1^wt^, adjusted for multiple comparisons.

Our previous study demonstrated that Bacteroidales antagonize one another using T6SS^iii^-dependent secretion of Tde (3). The MAG enrichment analysis (Fig. 1) suggested that families within Bacillota may also utilize Tde/Tdi for interbacterial antagonism. We obtained two Tde+ Bacillota donor strains, *E. quebecensis* DSM 23327 and *B. thuringiensis* ATCC 10792, that encode Tde/Tdi pairs adjacent to T7SS (Fig. 2A). Neither strain has been genetically modified in prior research. The genetically tractable recipient *B. thuringiensis* HD73 was antagonized by both Tde+ donors. To confirm that this is due to Tde effectors, we expressed the cognate immunity proteins from an IPTG-inducible promoter integrated into the Bt HD73 genome via an integrative and conjugative element (ICE) (28, 29). Expression of *tdi* increased Bt HD73’s fitness in competitive growth experiments under contact-promoting conditions (Fig. 2B and C). BtTdi1 expression was cross-protective against *E. quebecensis* antagonism, in addition to the cognate effector-encoding Bt ATCC 10792. In contrast, EqTdi1 expression was only protective against *E. quebecensis* and not Bt ATCC 10792. EqTdi1 mutations S84E and H103S substituted the corresponding amino acids from BtTdi1 and significantly reduced protection against *E. quebecensis* antagonism and toxicity (Fig. 2C), implicating these residues in cross-species protection. Expression of EqTde1 under the control of a “no-leak” arabinose-inducible promoter was toxic to *E. coli* (Fig. 2D). Co-expression of EqTdi1 fused to maltose-binding protein to improve solubility was protective against EqTde1-mediated toxicity (Fig. S2). We conclude that some Bacillota utilize the toxic effector Tde for interbacterial antagonism and that Tdi expression is protective. Some Bacillota Tdi, such as BtTdi1, provide immunity against Tde-mediated attack from distantly related species.

### BtTde1 secretion does not require an active T7SS and is enhanced by essC deletion

**Fig 3:**
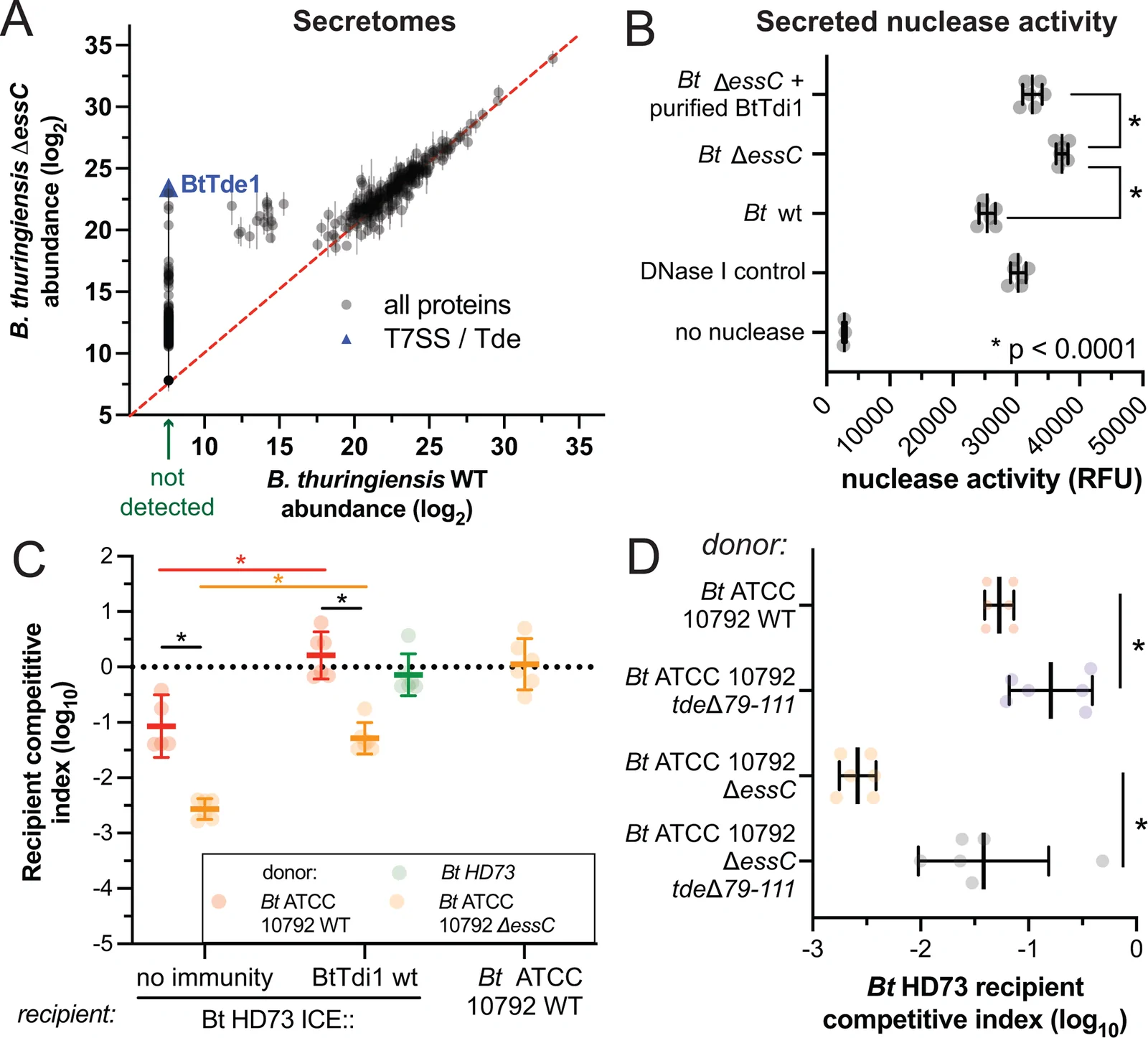
Deletion of *essC* enhances secretion of BtTde1 and antagonism. (**A**) Secreted proteins in Bt ATCC 10792 conditioned media were quantified with mass spectrometry. BtTde1 secretion was higher in the Δ*essC* background, whereas it was not detected in the wild-type strain. No other T7SS locus proteins were identified. (**B**) Nuclease activity in Bt ATCC 10792 supernatants, quantified with a fluorogenic DNA substrate, was increased in Δ*essC* and reduced by addition of purified BtTdi1. (**C**) Bt ATCC 10792 Δ*essC* showed enhanced antagonism of Bt HD73, which was reduced by expression of BtTdi1 in the recipient. BtTdi1 expression in the recipient and deletion of *essC* in the donor did not produce growth defects. Competitive indices with the parental strains were ~1. *P < 0.05, Student’s *t*-test. (**D**) In competitive growth experiments, inactivation of BtTde1 by deleting amino acids 79–111 resulted in a competitive disadvantage, both in the wild-type and the *essC* deletion backgrounds.

We asked whether BtTde1 is secreted by T7SS. Although the genomic proximity is suggestive, the interposed mobile element-related genes raise the possibility that BtTde1 is not a T7SS effector (Fig. 2A). The T7SS in *Bt* ATCC 10792 was inactivated by deleting *essC*, the conserved ATPase motor for secretion. Comparison of secreted proteomes in conditioned media from ∆*essC* and parental strains showed that BtTde1 abundance was near the median of all secreted proteins in ∆*essC* but undetectable in the wild-type strain (Fig. 3A). No other T7SS locus proteins were identified in secretomes. Secreted nuclease activity was also compared between these isogenic strains using a fluorescent nucleotide substrate (DNase Alert; Fig. 3B). Total secreted nuclease activity was higher in the Δ*essC* strain and was partially inhibited by the addition of purified BtTdi1 to conditioned media, indicating some BtTde1-specific activity. We conclude that BtTde1 secretion is enhanced by the deletion of *essC*, and an active T7SS is not required.

We hypothesized that *Bt* ATCC 10792 Δ*essC* would exhibit enhanced Tde-mediated antagonism. Indeed, deletion of *essC* provided a competitive advantage to *Bt* ATCC 10792 donors over Bt HD73 (Fig. 3C). However, immunity was conferred by expressing BtTdi1 in Bt HD73 or by the cognate BtTdi1 in *Bt* ATCC 10792 WT. We attempted to delete *bttde1* and *bttdi1* from *Bt* ATCC 10792 but were unable to achieve the allelic exchange. Manipulation at this site may be technically challenging due to the adjacent mobile element genes or other factors. However, we were able to delete in-frame an internal region of *bttde1* corresponding to amino acids 79–111. This region includes the active site and other structurally important residues. This mutation will eliminate activity and likely result in a misfolded product. The *bttde1*Δ79-111 mutation reduced competitive antagonism of Bt HD73 recipients in both the WT and Δ*essC* backgrounds (Fig. 3D). The effect size was larger for the Δ*essC* mutant, consistent with its enhanced Tde secretion.

We conclude that BtTde1 is secreted and does not require active T7SS for its secretion. In fact, deletion of *essC* enhances BtTde1 secretion and Tde-dependent antagonism of Bt HD73 recipients. The expression of BtTdi1 in recipients is protective against antagonism.

### Crystal structures of three Bacillota Tde/Tdi complexes demonstrate a conserved immunity mechanism

**Fig 4:**
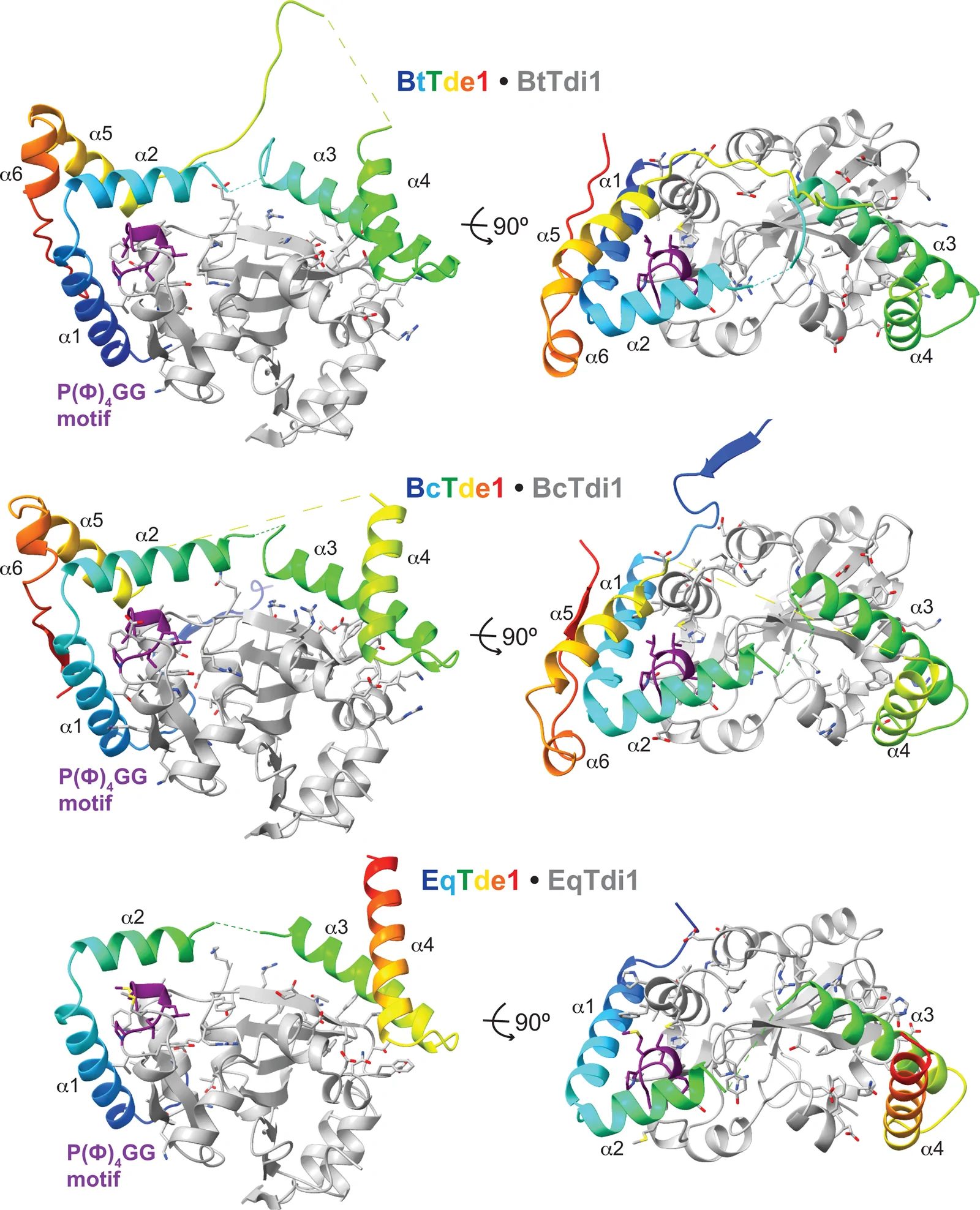
Crystal structures of three Bacillota Tde/Tdi complexes. Immunity and Tde Ntox15 domain complexes from *B. thuringiensis* ATCC 10792, *B. cereus*, and *E. quebecensis* are compared using Tdi-aligned structures. Tdes (rainbow colors for N- to C-terminus) are split into two subdomains, and the HxxD active site is disrupted. EqTde differs in its lack of ordered α5-α6 helices. Tdis form two interaction surfaces with Tde: a very highly conserved P(Φ)_4_GG motif and a less conserved interface with α3–α4 that is dominated by polar interactions.

Our previous study of T6SS-secreted Bacteroidaceae Tde identified an immunity mechanism that involves structural disruption of the effector with a large conformational change (3). We asked whether this mechanism is conserved in Bacillota. Five Bacillota Tde·Tdi pairs were selected for structural studies, including BtTde1·BtTdi1 and EqTde1·EqTdi1 based on the functional data (Fig. 2 and 3), as well as homologs from *Bacillus cereus*, *Tyzzerella nexilis*, and *Listeria boorei* to sample the sequence phylogenetic diversity and focus on accessible and potentially genetically tractable strains. Three were co-crystallized for structure determination by X-ray diffraction (Table S1 and Fig. 4). Sequence identity among these effector/immunity complexes ranged 64–73% in Tde and 50–56% in Tdi (Table S2). The overall structures are highly conserved (Fig. 4). However, the Tde C-terminus, including helices α5–α6, was disordered in the EqTde1·EqTdi1 structure. The structural similarity of the complexes was quantified using FoldSeek (30), and multimer TM-scores ranged 0.44–0.70 (Table S2). We also compared the new Bacillota Tde·Tdi structures to two complexes from *Phocaeicola vulgatus* (3). The BtTde1·BtTdi1 structure was similar to the *P. vulgatus* complexes (TM-score 0.57–0.61) despite 35–39% sequence identity, while the other two Bacillota complexes were more distant from *P. vulgatus* (TM-score 0.38–0.41, Table S2).

All three Tde·Tdi complexes exhibit a conserved mode of interaction, in which Tde is split into two subdomains with separate Tdi interfaces. The first interface involving Tde α3–α4 forms predominantly polar interactions between residues that are not highly conserved in either Tde or Tdi. At the second interface, the Tde α1–α2 and α5–α6 helices contact a very well-conserved Tdi P(Φ)_4_GG motif that inserts into the 4-helix bundle (Fig. S3). The P(Φ)_4_GG motif forms a short 3_10_ helical-type secondary structure with a conserved and unusual bifurcated hydrogen bond between the backbone carbonyl oxygen of Φ_1_ and the backbone amide nitrogens of Φ_4_ and the second conserved glycine, corresponding to positions i + 3 and i + 5 (Fig. S3). A second candidate bifurcated hydrogen bond is modeled from the Φ_2_ carbonyl to the first glycine amide (i + 3) and a conserved water (Fig. S3). A third backbone hydrogen bond occurs between Φ_2_ and the first conserved glycine (i + 3). In all three Bacillota Tde·Tdi structures, a tandem asparagine and arginine (N36 and R37 in BtTde1) participate in the H-bond network at the backbone carbonyls of Φ_3_ and the first conserved glycine (G152) (Fig. S3).

To more fully characterize Tde·Tdi complexes in gut bacteria, we took a structural bioinformatics approach using AlphaFold 3 (31) models of 231 Tde·Tdi pairs identified in MAGs. All Tde·Tdi models were compared pairwise using a FoldSeek multimer TM-score (30), and pairwise phylogenetic distances were calculated for both Tde and Tdi (32). Among these many structures, Tde·Tdi complex similarity was strongly related to Tdi sequence similarity but not Tde similarity or taxonomic distance (Fig. S4). We conclude that Tdi neutralizes Tde through a structural rearrangement and active site disruption mechanism that is conserved in Bacteroidota and Bacillota despite substantial sequence diversity. Predicted Tde·Tdi complex structural variability is primarily driven by the Tdi sequence.

### EqTdi1 binding promotes EqTde1 C-terminus mobility and sensitivity to proteolysis

The crystal structures (Fig. 4) have substantial disordered regions in Tde, including the active site and most of the DNA-binding site (Fig. S5) (3). In the case of EqTde1, the C-terminal α5 and α6 helices are also disordered (Fig. 4). We sought to differentiate crystallographic disorder artifact and actual Tde flexibility in solution. An inactivated mutant of EqTde1 (H279A/P281A) was labeled with ^15^N and used to generate ^15^N/^1^H HSQC NMR spectra (Fig. S6A). The addition of MBP-EqTdi1 resulted in the disappearance of most cross-peaks of ^15^N-EqTde1 due to binding of MBP-EqTdi1 to EqTde1 and the formation of a fairly large complex (~90 kDa). However, a subset of peaks remained strong, including seven glycine residues and three arginine side chains, which resonate in distinct spectral regions, indicating that these peaks belong to a protein segment that is highly mobile in solution. To confirm the high mobility of these detectable backbone amides in the ^15^N-EqTde1/MBP-EqTdi1 complex, we also performed ^15^N-heteronuclear NOE experiments, which showed that these backbone amides display near-zero NOE values, demonstrating that these amides/residues are indeed highly mobile in solution in the ^15^N-EqTde1/MBP-EqTdi1 complex. Thus, this highly mobile segment contains a Gly-rich sequence. Sequence analysis shows that EqTde1 has a Gly-rich C-terminal segment (after the HXXD active site). We conclude that the EqTde1 C-terminus is highly mobile in the EqTde1/EqTdi1 complex in solution.

Because Tdi binding to Tde results in C-terminus mobility, we hypothesized that Tde/Tdi complexes may be more susceptible to proteolysis. In limited trypsin proteolysis experiments (Fig. S6B), additional proteolytic fragments were observed for the EqTde1·MBP-EqTdi1 complex compared to either component alone. Because EqTde1 has C-terminal mobility while EqTdi1 exhibited minimal structural change upon binding, we hypothesized that these fragments are derived from EqTde1. Both fragments were identified by mass spectrometry as EqTde1, sharing a dominant peptide from the N-terminus (a.a. 48–59). Thus, the formation of the complex likely exposes mobile regions susceptible to trypsin proteolysis. The EqTde1·EqTdi1 crystal structure is unique, with its disordered C-terminal α5 and α6 helices. We are cautious about the generalizability of these findings to other Tde/Tdi pairs.

### Tdi inhibits Tde by mimicking a conserved active site motif

**Fig 5:**
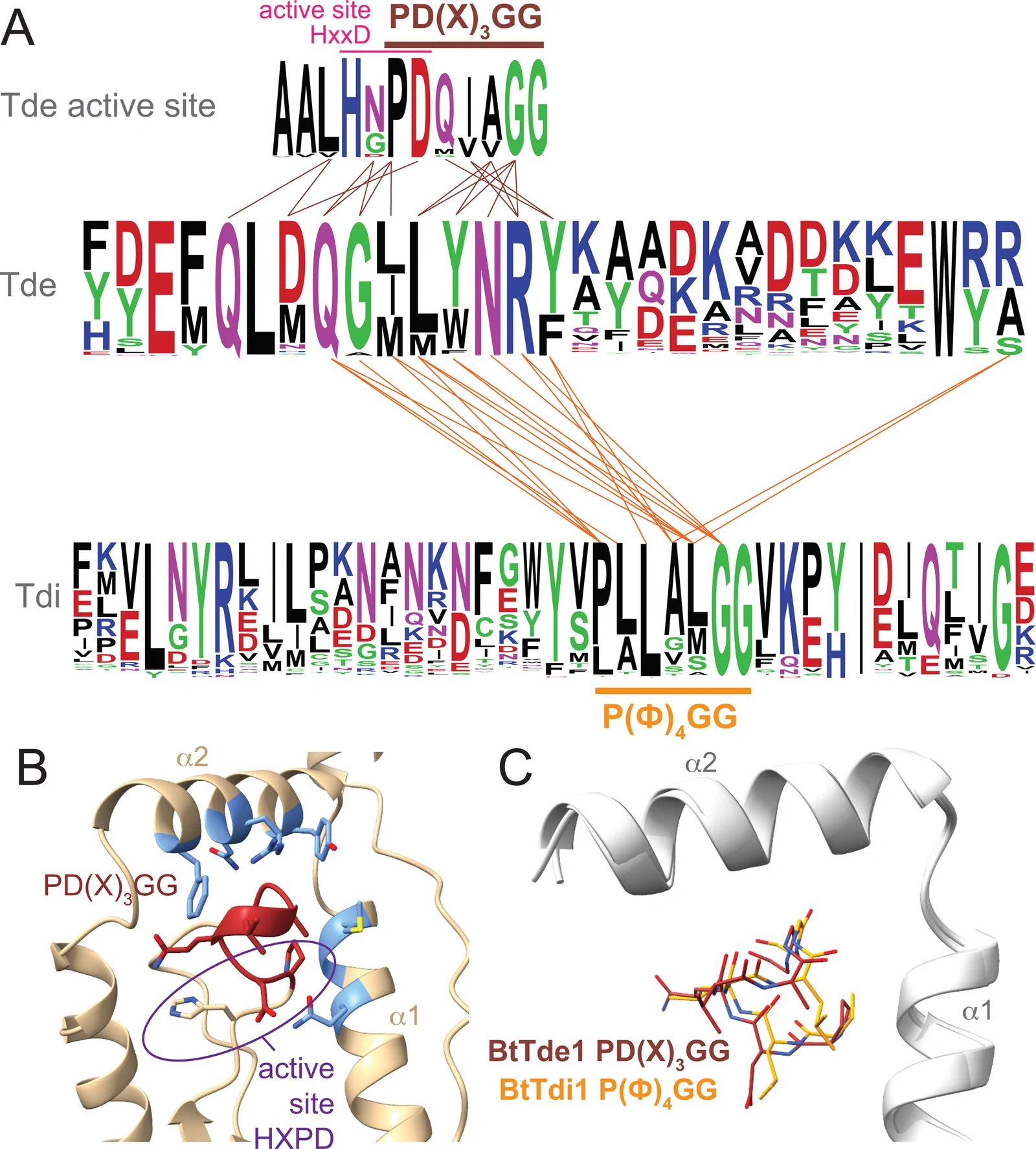
Immunity by active site motif mimicry is conserved in gut bacteria. (**A**) Using multiple sequence alignments of Tde and Tdi from MAGs, motifs are drawn for the Tde active site and all Tde/Tdi residues interacting at the effector/immunity interface. The very highly conserved Tdi motif P(Φ)_4_GG (orange) contacts many of the same highly conserved Tde residues that form intramolecular interactions with the Tde active site motif, which has a similar PD(X)_3_GG motif (brown). (**B**) An Alphafold3 model of *B. thuringiensis* Tde1 alone highlights the PD(X)_3_GG motif (red) interacting extensively with α1–α2 (blue). (**C**) Structures of BtTde1 alone (from panel **B**) and in complex with BtTdi1 were aligned by the α1 and α2 helices of Tde. The P(Φ)_4_GG motif of Tdi adopts a similar structure to the Tde active site PD(X)_3_GG motif.

To understand the amino acid sequence determinants underlying Tde neutralization, we aligned 200 unique, MAG-derived Tde/Tdi pair sequences and generated logos for the Tde and Tdi interface residues (Fig. 5A) (33). The Tdi P(Φ)_4_GG motif residues form extensive contacts with α1–α2 on Tde residues that are highly conserved, such as the 100% conserved tandem asparagine and arginine mentioned above, which form hydrogen bonds with the P(Φ)_4_GG peptide backbone (Fig. S3). In contrast, interactions at Tdi’s interface with the other Tde subdomain involve much less well-conserved sequences, suggesting that this interface may be important for Tde·Tdi binding specificity.

The α1–α2 surfaces of Tde that interact with Tdi in neutralized complexes surround the active site of Tde alone, as illustrated by an AlphaFold3 model of BtTde1 (Fig. 5B), which is highly similar to our previous crystal structure of *P. vulgatus* Tde1 (3). Interestingly, the active site of Tde has a 100% conserved PD(X)_3_GG motif that overlaps with the catalytic histidine and aspartic acid (HXXD motif) residues required for DNase activity (3). The PD(X)_3_GG motif makes extensive intramolecular contacts with α1–α2. A structural comparison of Tde’s PD(X)_3_GG and Tdi’s *P*(Φ)_4_GG motif in relationship to Tde α1–α2 (Fig. 5C) demonstrated striking structural similarity (mean Cα distance 0.95 Å), including the 3_10_ alpha helix architecture. We conclude that the P(Φ)_4_GG motif conserved in Tdi displaces the Tde active site that contains an equally uniformly conserved PD(X)_3_GG, upon assembly of the Tde·Tdi complex. Tdi’s structural mimicry of the Tde active site is likely required for neutralizing DNase activity.

### Two Tdi surfaces separately mediate binding affinity and thermal stability with Tde, both of which are required for neutralization of DNase activity

**Fig 6:**
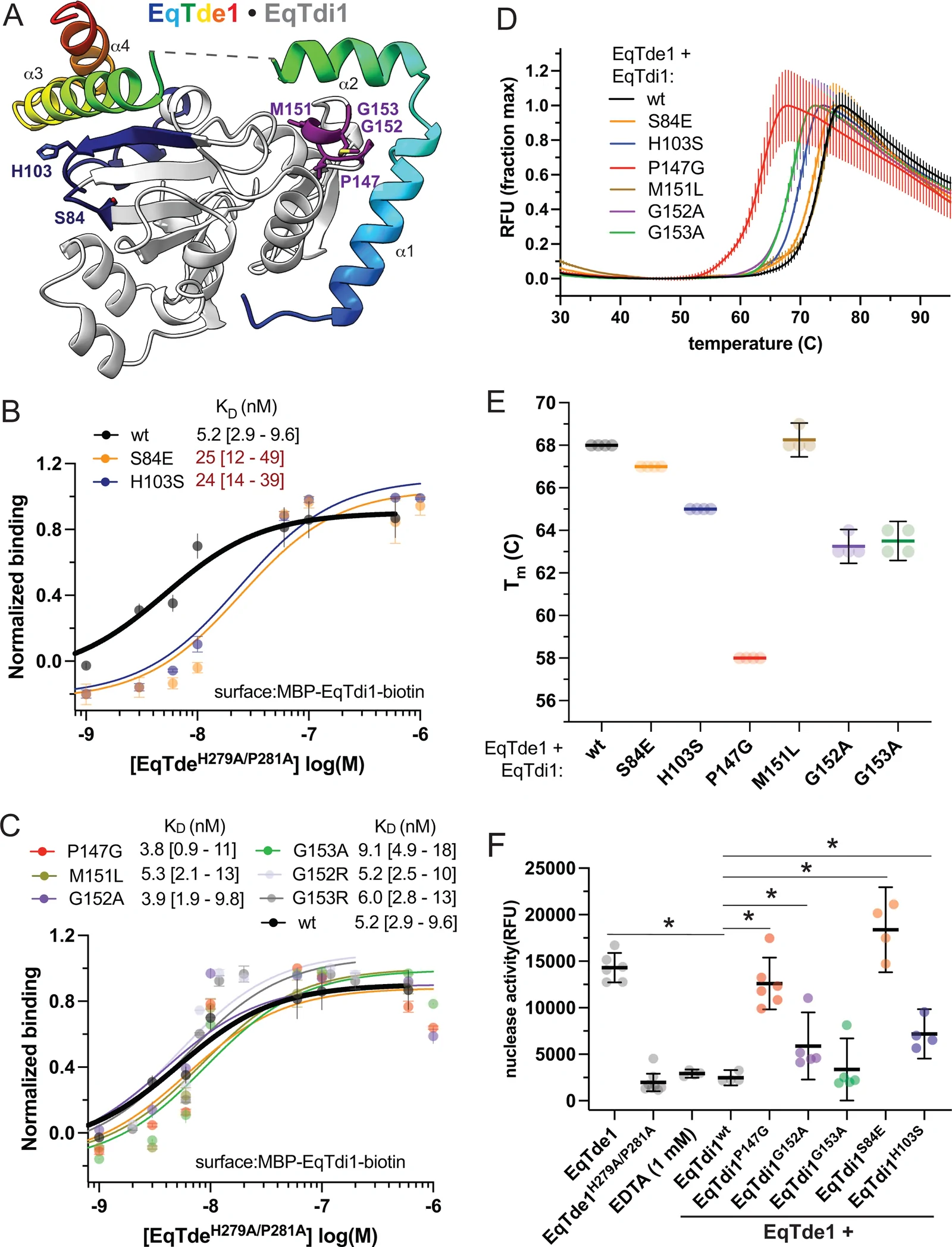
Tdi mutagenesis reveals distinct contributions of two Tde interface surfaces. (**A**) The target P(Φ)_4_GG motif and polar interface residues are highlighted on the crystal structure model of the EqTde1/EqTdi1. (**B**) Mutations at the EqTdi1 polar interface to the corresponding BtTdi1 residues, S84E and H103S, decreased binding affinity for EqTde1 as measured with biolayer interferometry. (**C**) EqTdi1 P(Φ)_4_GG motif mutations did not significantly affect affinity. Values in brackets are 95% confidence intervals, and red text indicates a significant difference from wild type. (**D and E**) EqTde1/EqTdi1 complex thermal stability was reduced by P(Φ)_4_GG motif mutations except for M151L. Smaller magnitude but significant reductions in thermal stability were observed for S84E and H103S. (**F**) Purified EqTde1 nuclease activity was neutralized by active site mutation, divalent cation chelation with EDTA, or addition of excess MBP-EqTdi1. All EqTdi1 mutants except G153A resulted in incomplete neutralization of EqTde1 nuclease activity.

To determine how the Tdi P(Φ)_4_GG motif and the separate polar interface with Tde contribute to effector neutralization, we mutated key residues of Tdi. EqTde1·EqTdi1 (Fig. 6A) was selected for detailed biochemical study because *Enterococcus* is prevalent in gut microbiomes, and the two components, including small amounts of the active EqTde1 toxin, could be recombinantly produced in *E. coli*. Mutation of EqTdi1 S84 and H103 at the polar interface with EqTde1 α3–α4 to the corresponding amino acids in BtTdi1 significantly reduced binding affinity from *K*_*D*_ ~5 nM to ~25 nM (Fig. 6B). Surprisingly, the very highly conserved residues of P(Φ)_4_GG were not required for high-affinity interaction with EqTde1 (Fig. 6C). To assess the effects of these mutations on the thermal stability of the Tde·Tdi complex, we co-purified EqTde1 in complex with wild-type or point-mutant EqTdi1 and performed differential scanning fluorimetry (Fig. 6D and E). All EqTde1·EqTdi1 complexes co-purified as heterodimers, confirming that none of these mutations markedly disrupts Tde/Tdi binding. All mutations of the EqTdi1 P(Φ)_4_GG motif, except for M151L (Φ_4_ position), reduced EqTde1·EqTdi1 thermal stability, most prominently P147G. The two mutations that lowered binding affinity (S84E and H103S) had significant but small-magnitude effects on thermal stability. We conclude that the Tdi polar residues interfacing with Tde α3–α4 mediate binding affinity and likely cross-species selectivity. Both this polar interface and the P(Φ)_4_GG motif contribute to the thermal stability of the Tde/Tdi complex.

We tested the requirement of EqTdi1 interface residues for the inhibition of the DNase activity of EqTde1, using a fluorogenic DNA substrate. EqTde1, purified under denaturing conditions and refolded, had DNase activity that was inhibited by active site mutation (H279A/P281A), chelation of divalent cations by EDTA, or addition of a 10-fold molar excess of EqTdi1 (Fig. 6F). EqTdi1 mutants P147G and S84E did not significantly inhibit effector nuclease activity, G153A fully inhibited, and G152A and H103S showed partial inhibition. We conclude that both the polar α3–α4 interface and P(Φ)_4_GG motif residues are necessary for full EqTde1 nuclease activity inhibition.

### A P(Φ)_4_GG motif peptide is sufficient to inhibit Tde at high concentrations

**Fig 7:**
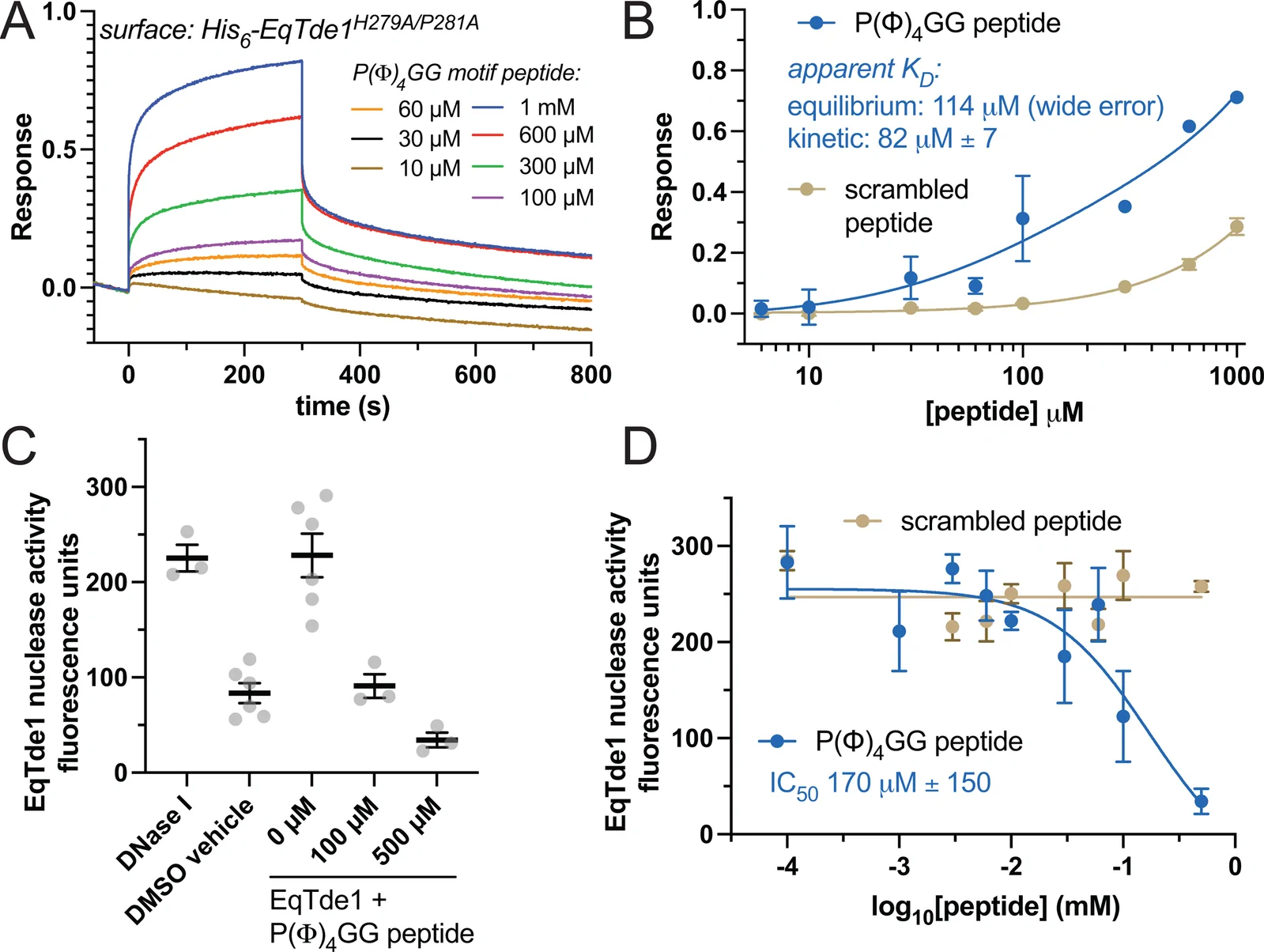
A P(Φ)_4_GG motif peptide is sufficient to bind and inhibit EqTde1 nuclease activity. (**A and B**) Peptide analyte binding to immobilized, catalytically inactive EqTde1 was measured using biolayer interferometry. Apparent affinities are low because the peptide must competitively displace the PD(X)_3_GG motif from the EqTde1 active site. Non-specific binding to the P(Φ)_4_GG peptide is less likely because of differences from a scrambled sequence peptide control. (**C and D**) The peptide was used to inhibit DNase activity of refolded wild-type EqTde1, measured with a fluorescent oligonucleotide substrate. In panel **C**, EqTde1 concentration is fixed, but P(Φ)_4_GG peptide concentration is increased during titration. Similarly high concentrations of peptides were needed to inhibit EqTde1, which has a similar level of DNase activity to bovine pancreas-derived DNase I. The scrambled peptide was inactive.

We hypothesized that Tdi P(Φ)_4_GG displaces the PD(X)_3_GG motif from the Tde active site to inhibit its nuclease activity. We synthesized a 14-residue peptide corresponding to EqTdi1’s P(Φ)_4_GG, with three and four additional amino acids on the N- and C-termini, respectively, and biotinylated on the N-terminus. Since the P(Φ)_4_GG competes with the covalently linked (high local concentration) PD(X)_3_GG motif of EqTde1, we anticipated that high excess concentrations of peptide would be needed to observe binding and enzymatic inhibition (Fig. S7). In support of this idea, binding was detectable by BLI only when EqTde1^H279A/P281A^ was immobilized, and excess peptide was added as the analyte (Fig. 7A; apparent *K*_*D*_ ~100 µM). No significant interaction was observed with streptavidin-immobilized peptide and EqTde1^H279A/P281A^ as the analyte or in fluorescence polarization assays with FITC-labeled peptide (Fig. S7). Because H279 is adjacent and P281 is a key conserved PD(X)_3_GG motif residue, the EqTde1^H279A/P281A^ inactive mutant may have different interactions with the Tdi P(Φ)_4_GG motif peptide than the wild-type protein. To address this possibility and test the ability of the peptide to inhibit enzymatic activity, we utilized refolded wild-type EqTde1 and a fluorogenic substrate DNase activity assay. High concentrations of P(Φ)_4_GG motif peptide inhibited the DNase activity of wild-type EqTde1 with a similar apparent IC_50_ (Fig. 7C and D). To test specificity, we synthesized a peptide with randomized ordering of the P(Φ)_4_GG motif amino acids (scrambled). This peptide bound EqTde1 with at least an order-of-magnitude lower affinity and did not significantly inhibit EqTde1 DNase activity up to a concentration of 600 μM (Fig. 7B and D).

### P(Φ)_4_GG mutations destabilize EqTde1/EqTdi1 and perturb BcTde1/BcTdi1 complex structures

To determine the structural effects of mutating the P(Φ)_4_GG, we pursued crystal structures of EqTdi1 P147G, G152A, and M151L. All three mutants were co-expressed with affinity-tagged EqTde1 in *E. coli* and co-purified as a 1:1 Tde·Tdi complex. However, all three Tdi mutants crystallized alone, with EqTdi1 P147G and G152A crystallizing as Tdi only under multiple conditions (Fig. S1 and S7). In all mutant structures, the P(Φ)_4_GG motif did not form substantial crystal contacts and exhibited a very similar overall structure. Subtle local candidate H-bond differences from the wild-type EqTde1·EqTdi1 complex were observed (Fig. S8), particularly for P147G, but could be related to solvent exposure in the absence of Tde. In pursuit of a Tdi P(Φ)_4_GG mutant structure in complex with a Tde, we obtained a structure of BcTde1·BcTdi1 P151G (Fig. S8). This mutant complex had a nearly identical structure to the wild type (Cα r.m.s.d. 0.23 Å). Thus, the conservative substitution of proline for glycine in P(Φ)_4_GG did not prevent active site mimicry. The findings confirm that P(Φ)_4_GG mutants have retained local folding and high specific activity, pertinent to the biochemical studies (Fig. 6). Crystallization of the EqTdi1 mutants alone in the presence of the high-affinity partner EqTde1, and the lowered melting temperature of P(Φ)_4_GG mutant complexes (Fig. 6D) suggest destabilization of the Tde·Tdi interface.

**Fig 8:**
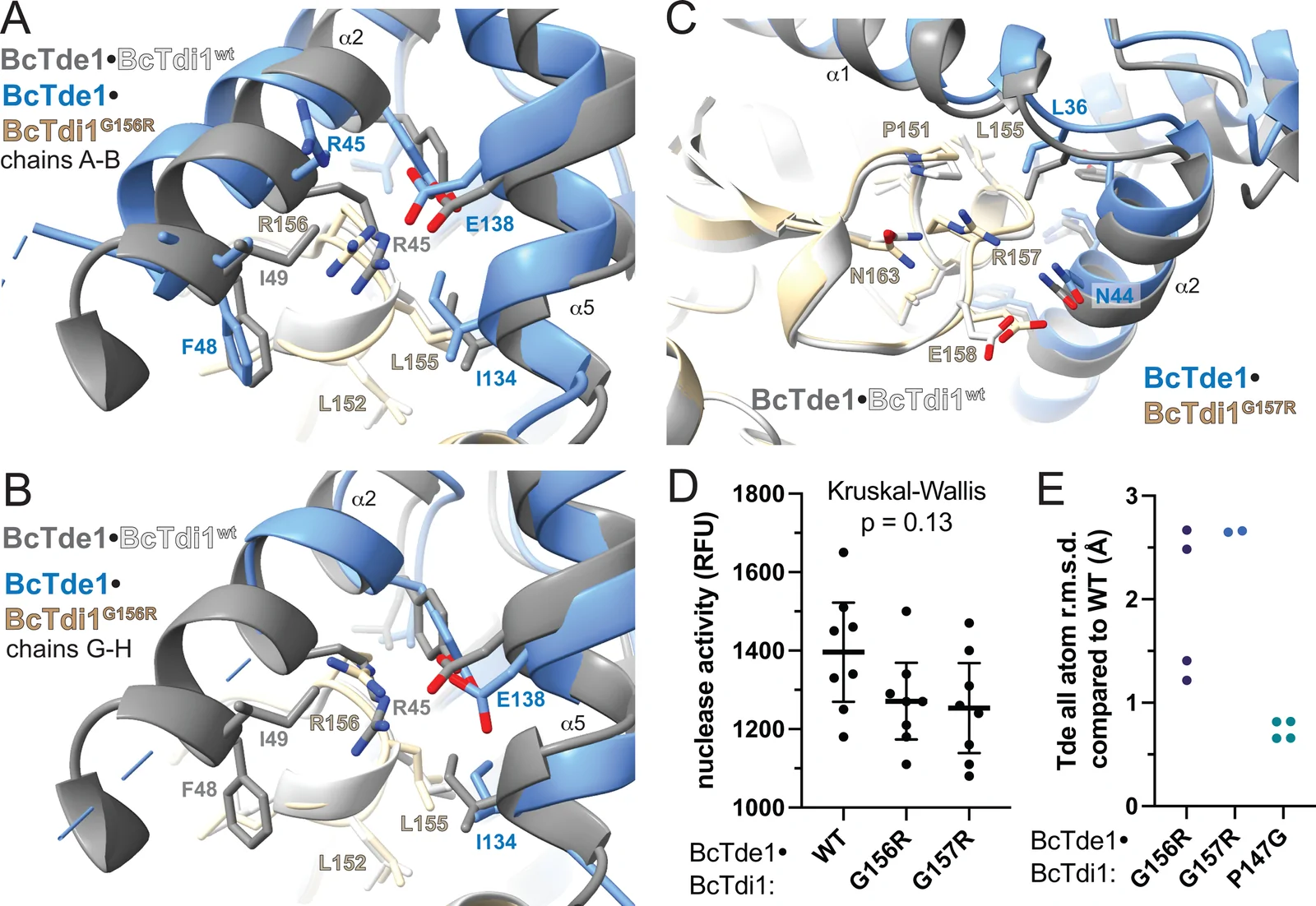
Non-conservative mutations of Tdi P(Φ)_4_GG perturb local structure while maintaining Tde nuclease inhibitory activi*ty.* (**A and B**) Structural models of two BcTde1·BcTdi1 G156R complexes from a single asymmetric unit are aligned to BcTde1·BcTdi1 WT. The bulky BcTdi1 mutant R156 side chain adopts a similar conformation to BcTde1 R45 in the wild-type structure. In the mutant structure, BcTde1 R45 is displaced with a different rotamer and a ~1 Å shift of the α2 helix. Within the four models of asymmetric unit, there is variability in BcTde1 structure to accommodate BcTdi1 R156. The adjacent part of the α2 helix is disordered in chains G–H, and α5 is shifted toward the C-terminus along its axis relative to the WT structure. (**C**) The mutant arginine of BcTde1·BcTdi1 G157R is accommodated by changes in adjacent BcTdi1 rotamers and a <1 Å shift of the conserved P151. BcTde1 α1–α2 and the intervening loop are modestly displaced. (**D**) The BcTde1·BcTdi1 complexes with the mutation of either conserved glycine exhibited no difference in fluorogenic DNA substrate nuclease activity compared to wild type. (**E**) BcTde1 structural differences are quantified with all-atom r.m.s.d. calculations after alignment of the WT and mutant BcTdi1 chains.

To more aggressively disrupt the *P*(Φ)_4_GG motif interaction with Tde, we crystallized and solved structures of non-conservative Tdi substitutions at the consecutive glycines in the *B. cereus* complex, BcTde1·BcTdi1 G156R and BcTde1·BcTdi1 G157R (Fig. 8). Both mutant complexes retained their overall Tde/Tdi interface structure, displaying plasticity of the local structure surrounding the inserted bulky arginine side chain. The mutant BcTdi1 R156 side chain displaced BcTde1 R45 from its position in the wild-type structure. The R156 residue was also accommodated by ~1 Å shifts of the α2 and α5 helices. Variations in the local BcTde1 structure at the mutant interface were observed among the 4 BcTde1·BcTdi1 complexes in the asymmetric unit (Fig. 8A and B). Mutation of the second conserved glycine in P(Φ)_4_GG, BcTdi1 R157, resulted in adjacent BcTdi1 side chain differences and a <1 Å shift of the conserved P(Φ)_4_GG proline 151 (Fig. 8C). The BcTde1 α1 and α2 helices were also shifted away compared to the wild-type structure to accommodate the R157 side chain. The overall BcTde1 structures in these BcTdi1 mutant complexes exhibit modest displacements of the peptide backbone and some local side chain rotamer differences, which were quantified as all-atom r.m.s.d differences from the wild-type structure in the 1–3 Å range (Fig. 8E).

We conclude that although the Tdi P(Φ)_4_GG motif is nearly 100% conserved and closely mimics the Tde active site, a remarkable structural plasticity at the Tde interface with this motif can accommodate non-conservative mutations.

### EqTdi1 requires polar interface residues, but not the full P(Φ)_4_GG motif, for protection against EqTde1 in competing bacteria

Non-conservative mutations in the nearly 100% conserved P(Φ)_4_GG perturb local structure but maintain the overall BcTde1·BcTdi1 interface. BcTdi1 G156R and G157R could each be co-expressed with active BcTde1 at high levels in *E. coli* during preparation for crystallography experiments, suggesting that these mutants maintain their ability to neutralize Tde. We measured the nuclease activity of BcTde1 in complex with either wild-type or mutant BcTdi1, finding no significant effects of G156R or G157R (Fig. 8D). EqTdi1-mediated protection from EqTde1 toxicity in *E. coli* overexpression experiments (Fig. 2D) required EqTdi1 G153, but not EqTdi1 P147 or G152, all of which are 100% conserved residues in P(Φ)_4_GG. As demonstrated by EqTde1·EqTdi1 complex co-purification and structural studies of BcTde1·BcTdi1 P151G (Fig. S8), the P > G Tdi mutant still forms a highly similar complex with Tde despite a substantial reduction in thermal stability (Fig. 6D). Polar interface EqTdi1 mutations S84E and H103S significantly reduced protection against EqTde1 toxicity (Fig. 2D). All EqTdi1 variants were expressed at comparable levels on Coomassie gels (Fig. S2A), excluding major reductions in expression as a likely explanation for loss of protection.

To test the requirements of P(Φ)_4_GG and the polar interface EqTdi1 residues for immunity against EqTde1-mediated interbacterial antagonism, we introduced a series of EqTdi1 mutants into the Bt HD73 recipient strain (Fig. 2C). Polar interface mutations to the corresponding BtTdi residues, S84E and H103S, significantly reduced the protective effects of EqTdi1 against competing *E. quebecensis*. The P(Φ)_4_GG motif G153A had reduced protection (Fig. 2C). However, other motif mutants, P147G and G152R, retained their protective effects, similar to EqTdi1 WT (Fig. 2C). EqTdi1 WT and G152R mutant expression levels in Bt HD73 were comparable, as measured with HiBiT peptide fusions and nanoluciferase complementation (Fig. S2B). However, mutant EqTdi1 expression was lower than WT, raising the possibility that some differences observed in mutant phenotypes across assays could be related to differential expression. We conclude that Tdi residues at the polar interface, which dominate binding affinity and specificity, are required for Tde neutralization and protection against antagonism. Although the Tdi P(Φ)_4_GG motif is nearly 100% conserved, point mutations at this interface have variable effects on Tde neutralization. Combined with the structural data, a likely explanation is that local Tde/Tdi P(Φ)_4_GG motif interface plasticity can accommodate point mutations without compromising effector neutralization under these conditions.

## DISCUSSION

### A model of Tde neutralization by binding, active site mimicry, and proteolytic degradation

**Fig 9:**
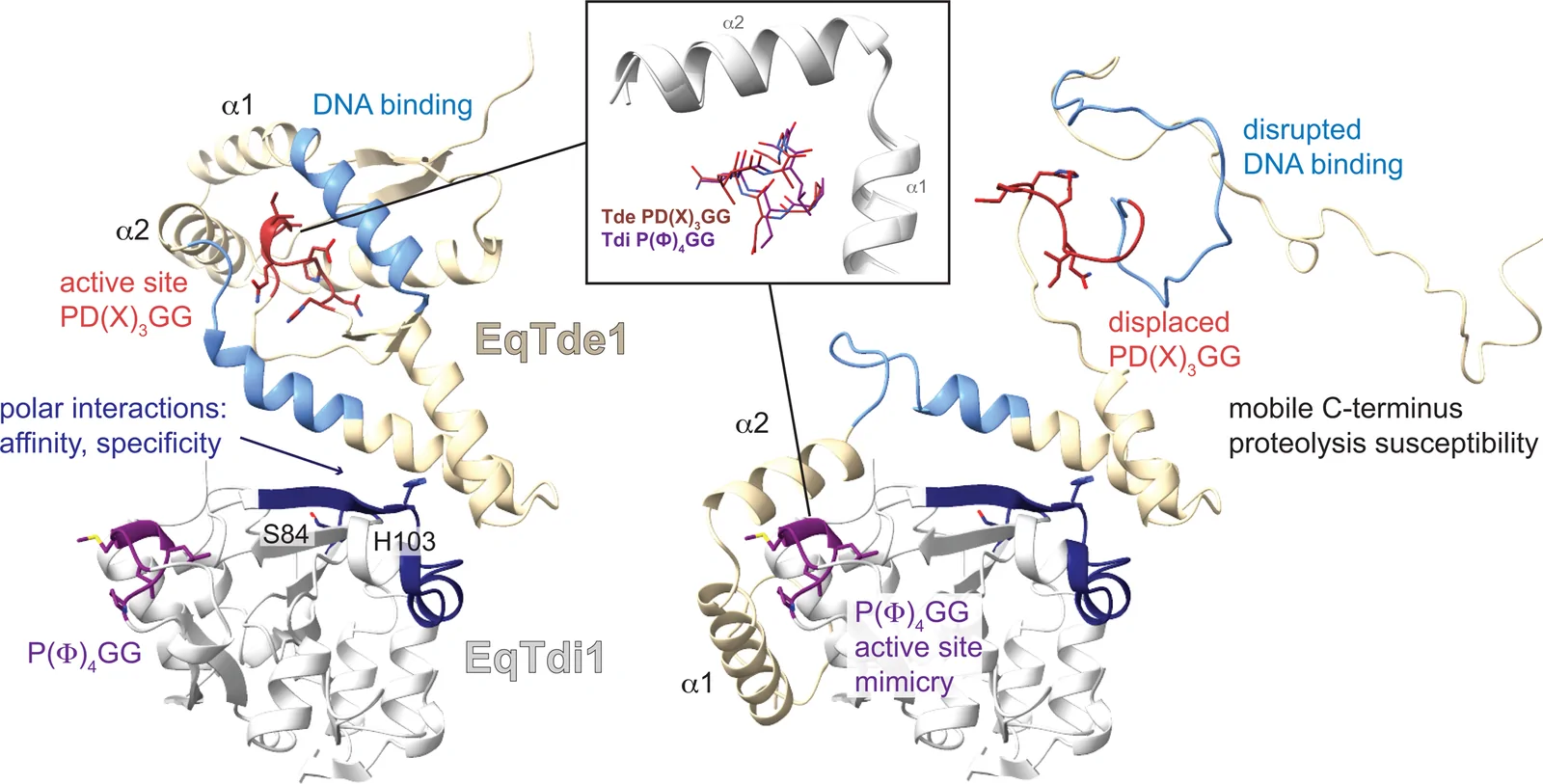
Summary model of EqTde1 neutralization by EqTdi1. (Left) Tde/Tdi affinity requires polar interactions (dark blue), including Tdi H103 and S84. EqTde1 is modeled with AlphaFold3 with a PD(X)_3_GG motif (red-brown) that overlaps with the active site. DNA-binding interfaces are adjacent to the active site (light blue). (Right) EqTde1 undergoes a large conformational change, forming a second interface with EqTdi at α1–α2. The EqTdi1 P(Φ)_4_GG motif (purple) mimics the EqTde1 active site, stabilizing this conformation. The C-terminus of EqTde including the active site and part of the DNA-binding region becomes mobile and susceptible to proteolysis. The C-terminus is disordered in the EqTde1/EqTdi1 crystal structure and is modeled with MODELLER.

We synthesized the structural and biochemical data into a model of Tde inactivation, illustrated with EqTde1·EqTdi1 (Fig. 9). The affinity and specificity of Tde/Tdi interaction are mediated by an initial binding interface at the immunity’s polar residues (H103, S84) and the α3–α4 helices of the effector. A high-affinity interaction involving this Tdi surface is required for DNase inhibition and for protecting the recipient against Tde. Upon binding, Tde undergoes a large conformational change. This conformational change is likely driven by free energy of binding, which is calculated to be 134 kcal/mol lower in the experimental EqTde1·EqTdi1 structure than in the modeled single interface initial interaction (Fig. 9). This hypothesis is supported by the thermal stability of EqTde1·EqTdi1 and destabilization by P(Φ)_4_GG motif mutagenesis (34). On the order of 10 kcal/mol is generally required for conformational changes in proteins (35).

Upon conformational rearrangement, the Tde helices α1 and α2 move away from the Tde active site PD(X)_3_GG motif, forming extensive contacts with the structural mimetic P(Φ)_4_GG motif of Tdi. This secondary interface is required for the stability of the Tde·Tdi complex. The Tde conformational change displaces the active site and disrupts the DNA-binding site. We hypothesize that point mutations of the P(Φ)_4_GG motif shift the equilibrium between these two Tdi-bound conformations of Tde. The biochemical experiments with supra-physiological concentrations of the isolated P(Φ)_4_GG motif peptide (Fig. 7) indicate that P(Φ)_4_GG alone is not sufficient to displace the Tde active site that is covalently attached to the effector. However, a high-affinity Tde⋅Tdi interaction at the polar interface increases the local concentration of Tdi’s P(Φ)_4_GG motif, allowing a multivalent interaction to efficiently displace the Tde active site. The effector C-terminus becomes structurally mobile (especially in the case of EqTde1), increasing the effector’s susceptibility to proteolysis. A limitation of this study is that we were unable to capture a structural snapshot of Tde·Tdi in the proposed state with an intact effector active site. Three structures of Tdi P(Φ)_4_GG motif mutants co-purified with Tde crystallized alone. A proline-to-glycine mutant, BcTde1·BcTdi1 P151G, had a structure essentially indistinguishable from wild type. The more disruptive mutations BcTde1·BcTdi1 G156R and G157R resulted in local structural perturbations to accommodate the bulky added side chain but retained a similar overall Tde·Tdi interface.

Effector active site mimicry, enabled by very high-affinity effector binding at a secondary interface, represents a new mode of polymorphic toxin effector neutralization. The majority of known immunity mechanisms are driven by steric occlusion of the effector’s active site, blocking access to critical enzymatic substrates. However, there is precedent for more elaborate mechanisms, such as the reversal of the effector’s enzymatic activity (7) and structural rearrangement, disrupting the effector’s activity (3, 9). This mode has potential advantages of efficiently eliminating toxic effector enzymatic activity and promoting rapid effector elimination through proteolytic pathways in the recipient bacterium.

The near 100% conservation of P(Φ)_4_GG in otherwise sequence-dissimilar Tdi from diverse bacterial gut MAGs indicates strong selective pressures for active site mimicry by this motif. A striking and unexpected finding in our mutagenesis studies was that the conserved proline and dual glycines were not strictly required for Tde binding or protection against Tde-mediated toxicity. The EqTdi1 mutant P147G lost its ability to inhibit purified EqTde1 nuclease activity *in vitro* but was still protective against antagonism and toxicity in *E. coli*. This could be due to several possibilities, such as retained steric hindrance of EqTde1 accessing its *in vivo* target (genomic DNA) in contrast to a short oligo substrate in the *in vitro* experiments, expression or stability differences in cells, or the sensitivity of the *in vivo* assay for nuclease activity-mediated toxicity. The structural studies of BcTde1⋅BcTdi1 P(Φ)_4_GG demonstrate flexibility at this Tde/Tdi interface to accommodate amino acid substitutions without large changes to the overall neutralized effector complex structure. Tdi P(Φ)_4_GG is not the major contributor to Tde binding affinity, and its sequence can be varied without immediate detrimental effects in our experimental systems. Limitations of our studies may contribute to partial or undetectable effects of P(Φ)_4_GG mutants. For example, overexpression of mutant Tdi in the prey strain during high-density competitive growth and *E. coli* toxicity experiments could compensate for suboptimal Tde neutralization activity. We speculate that the strict conservation of P(Φ)_4_GG is related to its contributions to Tde⋅Tdi stability, effectively “locking” Tde in an inactive state that becomes proteolytically susceptible after the inciting interaction at the polar interface (Fig. 9). Evidence for this includes thermal instability of the Tde⋅Tdi complex upon mutation of the P(Φ)_4_GG motif, crystallization of EqTdi1 P(Φ)_4_GG motifs without the co-purified EqTde1, and the structural variability among BcTde1⋅BcTdi1 G156R mutant complexes.

In the context of interbacterial antagonism, delivery of one or a few Tde molecules to the non-immune recipient cytoplasm is likely sufficient for genomic DNA damage and cell death. To be protective, Tdi must quickly bind and inactivate Tde and maintain the neutralized complex until Tde can be degraded. Our study suggests that P(Φ)_4_GG is most needed for the latter process. We hypothesize that P(Φ)_4_GG mutant overexpression in *E. coli* toxicity assays and ICE-engineered Bt HD73 recipient strains may compensate for mutant effects on Tde complex stability and turnover.

### Bacillota Tde/Tdi in the gut microbiome context

Interbacterial antagonism in the gut microbiome has primarily been studied in gram-negative bacteria, with a particular emphasis on Bacteroidales (13, 14, 16–18, 20). Our MAG analysis highlights the prevalence of polymorphic toxin effector and immunity pairs in gut gram-positive bacteria, including Tde/Tdi. A minority of the Bacillota *tde* we identified were found in organisms with a T7SS, including the *Enterococcus* and *Bacillus* model strains utilized in our study. Our investigation of the T7SS as a candidate secretion mechanism for BtTde1 in *Bt* demonstrates a limitation of this associative bioinformatic analysis, i.e., that an effector can be encoded in the same genome or even adjacent to a secretion system that is not its primary delivery mechanism. We considered the possibility that BtTde1/BtTdi1 are a toxin-antitoxin system, but several lines of evidence argue against this possibility. BtTde1 is secreted, it mediates antagonism with other bacteria, and BtTdi1 is highly stable. The secretion mechanism for BtTde1 is unknown and remains a focus of our future research in addition to potential counter-regulatory interactions with *essC*. Determining whether EqTde1 is secreted by the adjacent T7SS will require targeted inhibition or genetic modification, which we were unable to accomplish due to challenges with genetic tractability. Most *tde*-positive gut Bacillota, such as *Ruminococcus* MAGs, lacked an associated dedicated toxin secretion system. The strong association of *tde/tdi* with mobile elements and T4SS, including conjugative subtypes, suggests that T4SS may be a frequent mode of horizontal transfer of these elements. It is possible that some gut Bacillota use T4SS for Tde secretion or that Tde is delivered through an unknown secretion mechanism.

Substantial sequence variation among Tde and Tdi homologs was observed in gut MAGs, a pattern consistent with prior observations in polymorphic effector and immunity classes. This variability likely reflects the “arms race” resulting from the interbacterial antagonism advantages of secreting effectors that circumvent immunity-mediated inactivation and/or expressing immunity proteins that protect against many attacker effectors. Our study highlights sequence variability at the Tde/Tdi polar interface, which is the dominant interaction needed for high-affinity interaction and required for Tde neutralization. Although not the focus of this work, several of our findings suggest that the polar interface is a major determinant of specificity, i.e., of which Tde variants a Tdi can neutralize. For example, BtTdi1 can protect against donors wielding either BtTde1 or EqTde1, whereas EqTdi1 can only protect against the cognate effector (Fig. 2B). Point mutagenesis of EqTdi1 polar interface residues to the corresponding amino acids in BtTdi1 (S84E and H103S) reduced EqTde1 binding and neutralization in antagonism experiments. Thus, polar interface Tdi residues are required for interaction with and neutralization of specific Tde. Our structural bioinformatics study using AlphaFold modeling (Fig. S4) indicates that these sequence variations on the Tdi side of the interaction are a primary determinant of Tde/Tdi structures across MAGs.

In summary, this study provides detailed structural and functional insights into a polymorphic toxin effector and immunity protein family that mediates antagonism among diverse gut Bacillota. Future directions include identifying effector secretion mechanisms and determining Tde’s antagonistic effects within complex gut microbiome communities.

## MATERIALS AND METHODS

### Competitive growth assays

Bacillota strains were grown overnight at 37°C on LB or BHIS agar. Bacteria were resuspended in the corresponding broth, and the optical density of each strain was adjusted to achieve either a 10:1 or 1:1 donor-to-competitor ratio. Competitions involving *E. quebecensis* were plated on BHIS agar and incubated anaerobically for 24 h, while all other competitions were performed on LB agar under aerobic conditions for 24 h. For competitions involving strains in which immunity genes were cloned and integrated on an integrative and conjugative element (ICE), co-cultures were plated on LB agar supplemented with 1 mM IPTG under cell-cell contact–inducing conditions as described above. After incubation, cells were recovered in LB or BHIS broth, serially diluted, and plated with or without chloramphenicol to select for ICE-inserted strains of *B. thuringiensis*. Competitive indices for recipient strains were calculated using colony-forming units (CFU) as (post-competition recipient/pre-competition recipient) ÷ (post-competition donor/pre-competition donor). All experiments included at least three biological replicates and three independent repetitions. Statistical significance was determined using unpaired two-tailed *t*-tests.

### *Bacillus thuringiensis* genetics

BtTdi1, EqTdi1, and mutants were placed under the control of an IPTG-inducible promoter with a chloramphenicol resistance cassette on a plasmid that integrates into mini-ICE*Bs*1 when transformed into *Bacillus subtilis* XPORT strain JAB932 (29). All plasmids used in this study for immunity protein expression were derived from pCE697 (36). Transformed and integrated JAB932 cells were selected on Luria broth containing chloramphenicol and D-alanine (JAB932 cells are auxotrophic). The ICE containing EqTdi1 was then conjugated into *Bacillus thuringiensis* HD73, which has a conserved *attL* site for targeted insertion. Bt HD73 conjugants selected on Luria broth with chloramphenicol and without D-alanine were confirmed to have ICE insertion by PCR and Sanger sequencing.

Gene deletion by allelic exchange in *Bacillus thuringiensis* strains ATCC 10792 and HD73 was performed using the temperature-sensitive shuttle vector pMAD, which is broadly applicable in Bacillota (37). All pMAD plasmids were produced unmethylated in Dam⁻/Dcm⁻ competent *E. coli* to circumvent restriction enzymes in Bt. Bt was made electrocompetent by washing and resuspension in 40% PEG 6000. pMAD allelic exchange plasmids were delivered by electroporation at 25 µF, 400 Ω, and 2.5 kV in 0.2 cm gap cuvettes with 100 µL of electrocompetent cells, followed by immediate recovery in LB medium at 30°C for 3 h. Transformants were selected on Luria broth with 5 µg/mL erythromycin. The transformed strains were cured of plasmids at 40°C. Allelic exchange and gene deletion were confirmed using PCR and Sanger sequencing.

This study included metagenome-assembled genome bioinformatics, *Escherichia coli* toxicity, protein and peptide purification, crystallography, NMR spectroscopy, structure and sequence bioinformatics, limited trypsin proteolysis, differential scanning fluorimetry, biolayer interferometry, fluorescence polarization, nuclease activity, secretomes, mass spectrometry protein identification, and HiBiT expression quantification (see Supplemental text).

## Data Availability

All strains and plasmids used in this study will be made available upon request from qualified investigators to the corresponding author. All crystal structure data have been deposited in the PDB. Accession codes are listed in Table S1. Limited trypsin proteolysis band identification mass spectrometry data have been deposited in MassIVE (accession MSV000102581).

## SUPPLEMENTAL MATERIAL

Supplemental figures mbio.01516-26-s0001.pdf Figures S1 to S8.

Supplemental text mbio.01516-26-s0002.pdf Supplemental methods.

Supplemental tables mbio.01516-26-s0003.xlsx Tables S1 and S2.

ASM does not own the copyrights to Supplemental Material that may be linked to, or accessed through, an article. The authors have granted ASM a non-exclusive, world-wide license to publish the Supplemental Material files. Please contact the corresponding author directly for reuse.
