# Supplementary material for: Secreted nuclease effector neutralization by active site mimicry in Bacillota
Source: mBio. 2026 Aug 17;17(9):e01516-26. doi: 10.1128/mbio.01516-26 (PMC13556189; doi:10.1128/mbio.01516-26)
Supplement: Supplementary file 1 [file mbio.01516-26-s0001.pdf]

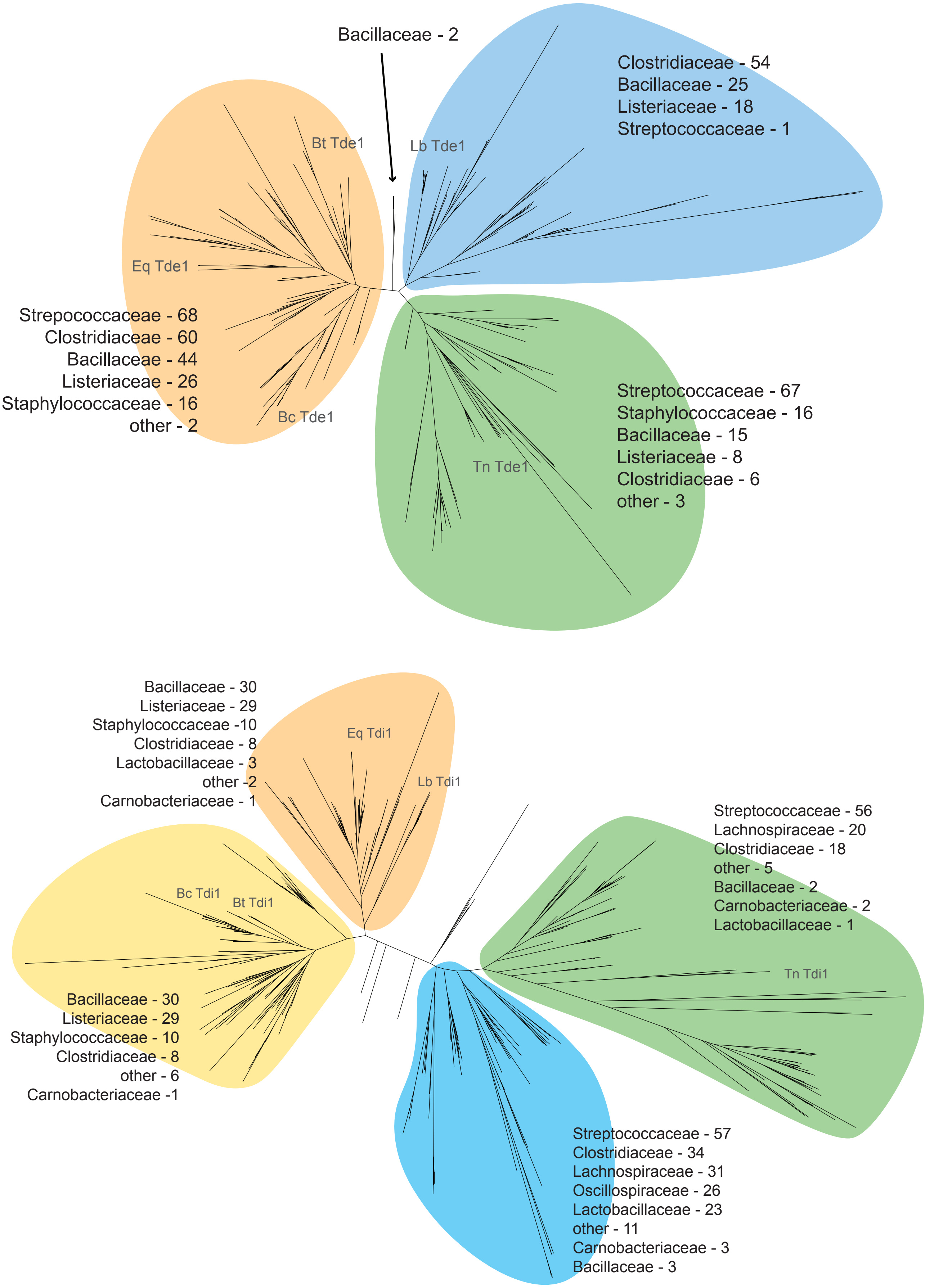

Figure S1. *Phylogenetic trees of Bacillota Tde and Tdi.* Bacillota with sequenced genomes in NCBI databases that encode *tde/tdi* pairs were identified with BLAST, aligned, and compared with radial dendrograms. Tde and Tdi are found in a greater diversity of Bacillota families than observed in gut MAGs. There is poor correlation of sequence similarity (arbitrary clades highlighted in color) and taxonomy. Representation of most families in each clade is highly suggestive of horizontal gene transfer events.

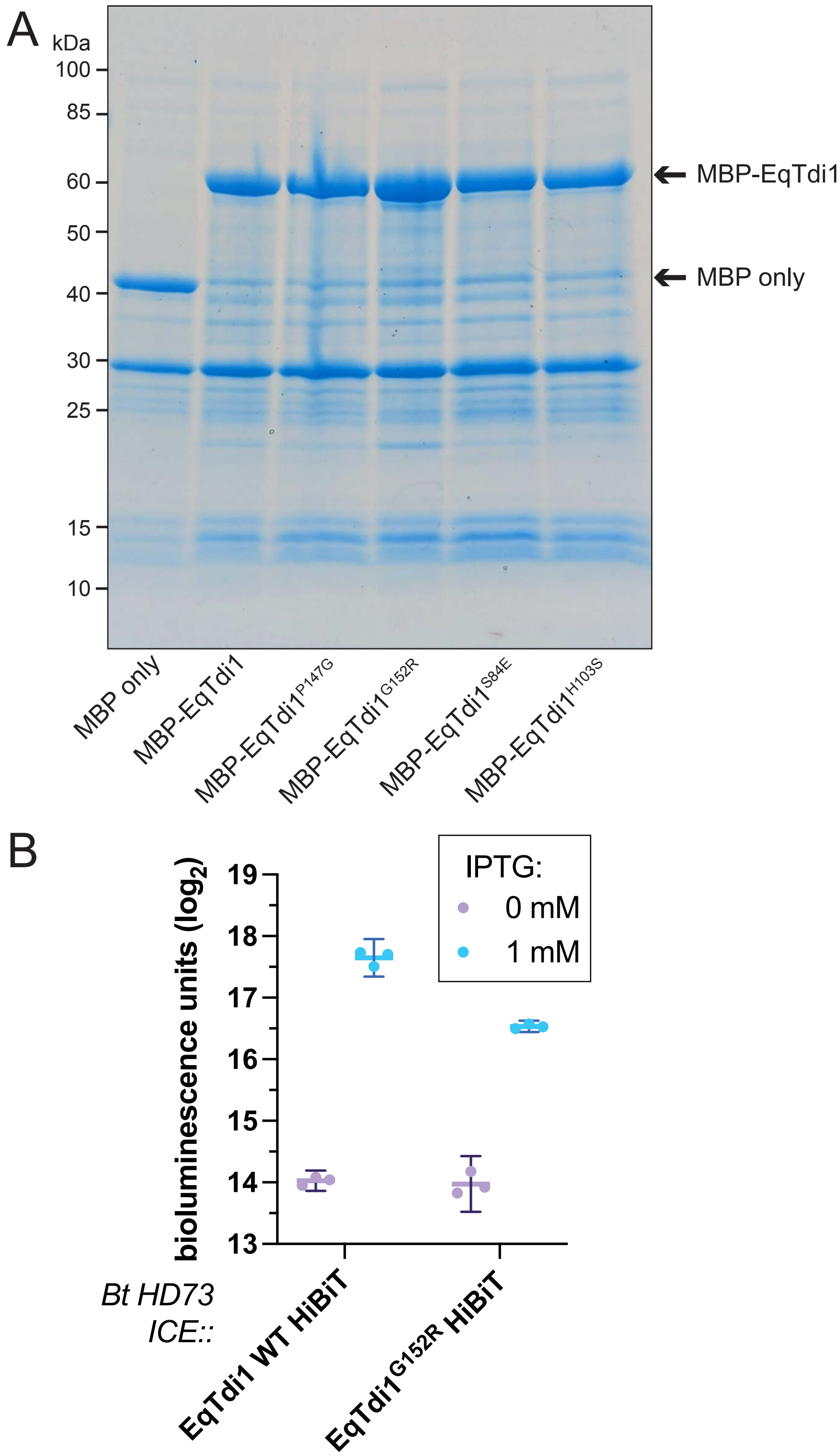

Figure S2. *EqTdi1 and mutants are expressed in E. coli toxicity and Bacillus antagonism experiments.* (A) Overexpressed MBP or MBP-EqTdi1 is detectable with Coomassie staining of lysates. EqTdi1 mutants are all expressed at comparable levels to WT. (B) Inducible expression in Bt HD73 was evaluated with C-terminal HiBiT fusions of EqTdi1 WT and one mutant.

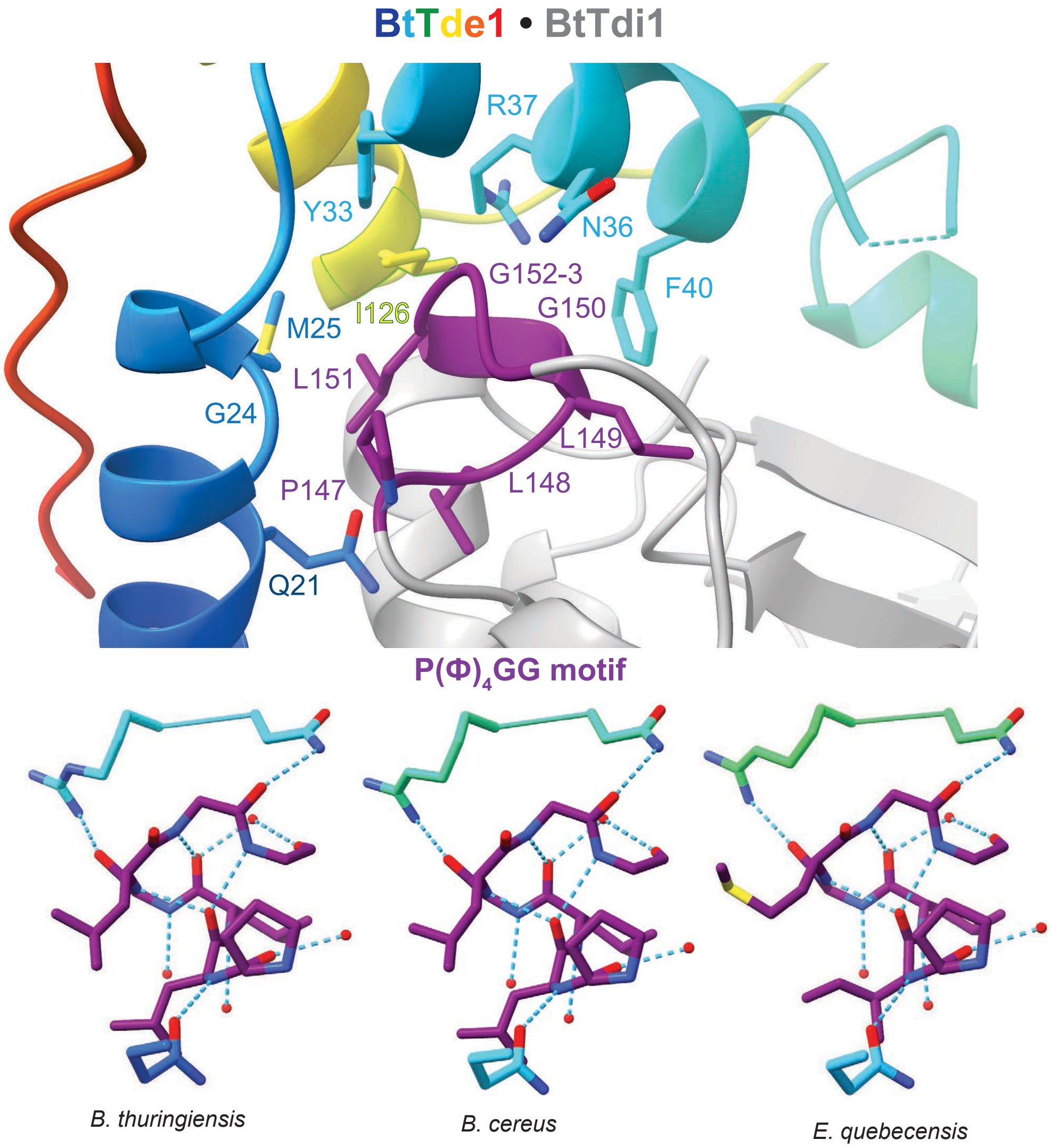

Figure S3. *BtTdi1 P(Φ)$_4$GG motif interactions with BtTde1 and hydrogen bonds.* The conserved Tdi motif (purple) forms a short helix at the protein surface, which forms extensive contacts with Tde. Tdi residues P147 and L151 have hydrophobic interfaces with Tde G24, M25, and I126. Conserved Tde residues N36 and R37 each form hydrogen bonds with the Tdi backbone at G152 and G150, respectively. The conserved aromatic, Tde F40, hydrophobically interfaces with the (Φ)$_4$ motif.

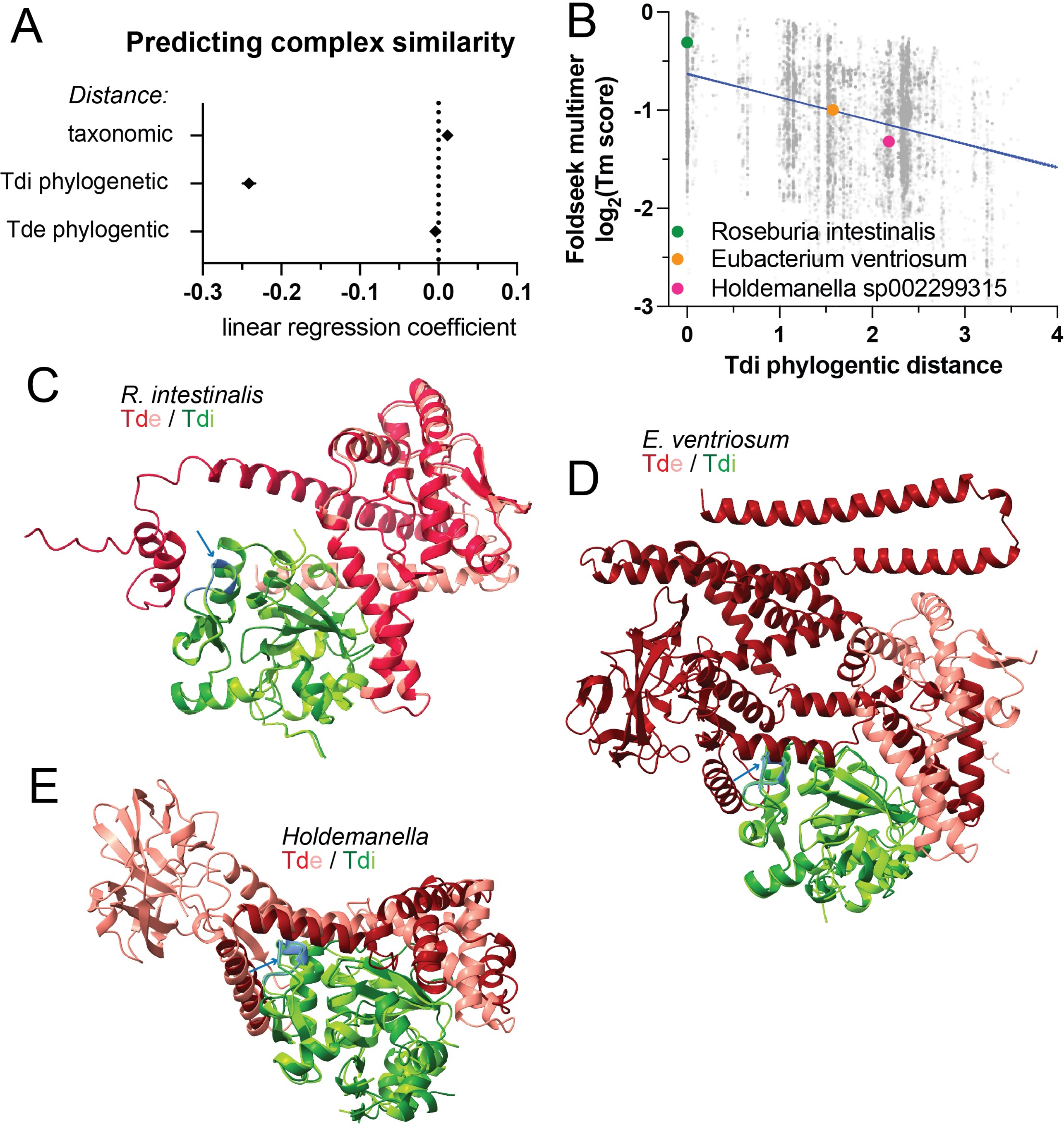

Figure S4. *Tdi phylogenetic distance correlates with Tde/Tdi structural differences.* 231 Tde/Tdi complexes were modeled using AlphaFold 3. (A) Linear regression models showed a dominant effect of sequence-based Tdi phylogenetic distance on the structural similarity of Tde/Tdi pairs. Tde sequence and taxonomic difference were not strongly correlated to structural similarity. (B) The relationship of Tdi phylogenetic distance to structural similarity is graphed. Distant Tdi sequences correlate with dissimilar Tde/Tdi structure. (C-E) Three Tde/Tdi structure pair models are overlayed with Tde in shades of red, Tdi in shades of green, and $P(\Phi)_4GG$ motif in blue. Tde/Tdi pairs were selected from MAGs of the same species (taxonomic distance = 0).

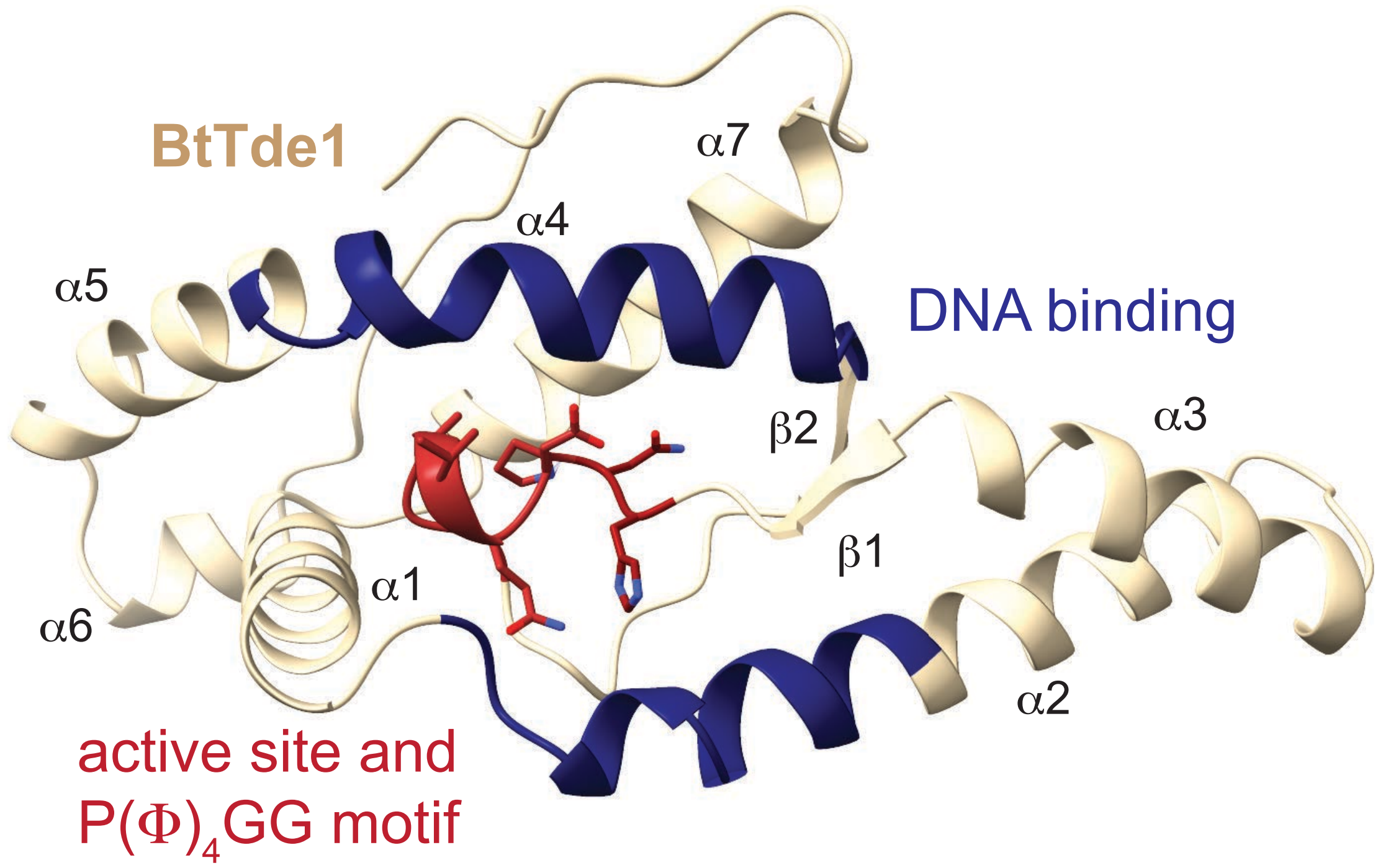

Figure S5. *A BtTde1 only AlphaFold model with predicted DNA-binding and active sites.* The BtTde1 structure is predicted with AlphaFold 3 and has high similarity to the experimental *P. vulgatus* Tde (PvTde1) structure. The predicted DNA binding site is colored dark blue, supported by prior mutagenesis studies in PvTde1. The active site HxPD motif and overlapping $P(\Phi)_4GG$ motif are modeled in dark red.

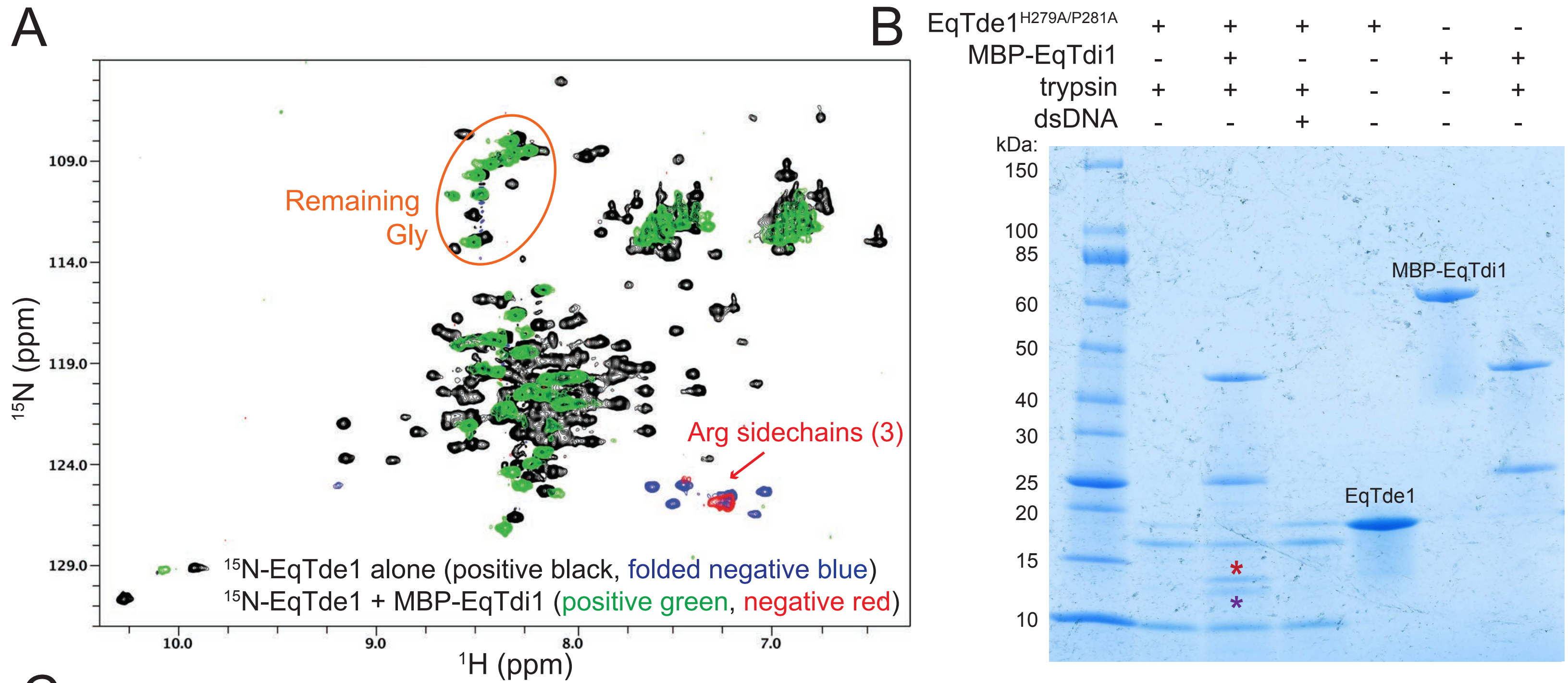

C

MEVSFKKNAKHDAEEFARQLKNQEKGMNELTVEEYLANRERYLAEGRALEGNIAQQAAREQAYTKKLNELQKSGKTLSQAKS

EAKQWLDKQAALANPAQIAGGKANIIGGMGDKGINSSLGSQWRYRIEAVDEQIRAMAKNMTPEQLKNTHLNVKLTQHHHHHH

Figure S6. *EqTdi1 binding results in mobility of the C-terminus and increased proteolytic susceptibility in EqTde1.* A) Overlays of ^{15}N/^{1}H HSQC NMR spectra of ^{15}N-labeled, catalytically inactive EqTde1$^{H279A/P281A}$ in the absence and presence (1:1 ratio) of unlabeled MBP-EqTdi are shown. Most of the cross-peaks disappeared due to the formation of a very large complex (~90 kDa). However, about 27 backbone amide cross-peaks (including 7 glycine residues with strong peak intensity) and 3 arginine sidechain peaks of ^{15}N-EqTde1 remain detectable, suggesting that these residues are highly mobile even in the ^{15}N-EqTde1/MBP-EqTdi1 complex. EqTde1 has a glycine-rich C-terminal segment that is highly mobile in the EqTde1/EqTdi1 complex. Moreover, the C-terminal EqTde1 contains 3 arginine residues, consistent with the 3 detected Arg sidechain resonances in the complex. B) Limited trypsin proteolysis performed on EqTde1$^{H279A/P281A}$, MBP-EqTdi1 and the 1:1 complex highlighted additional proteolytic products (asterisk) upon EqTde1 interaction with Tdi. dsDNA substrate did not affect EqTde1 proteolysis. C) EqTde1 coverage was mapped for the three most abundant peptides identified by mass spectrometry for the upper (red) and lower (purple) protein bands marked by asterisks in panel B. The dominant peptide (bold line) was present in both fragments.

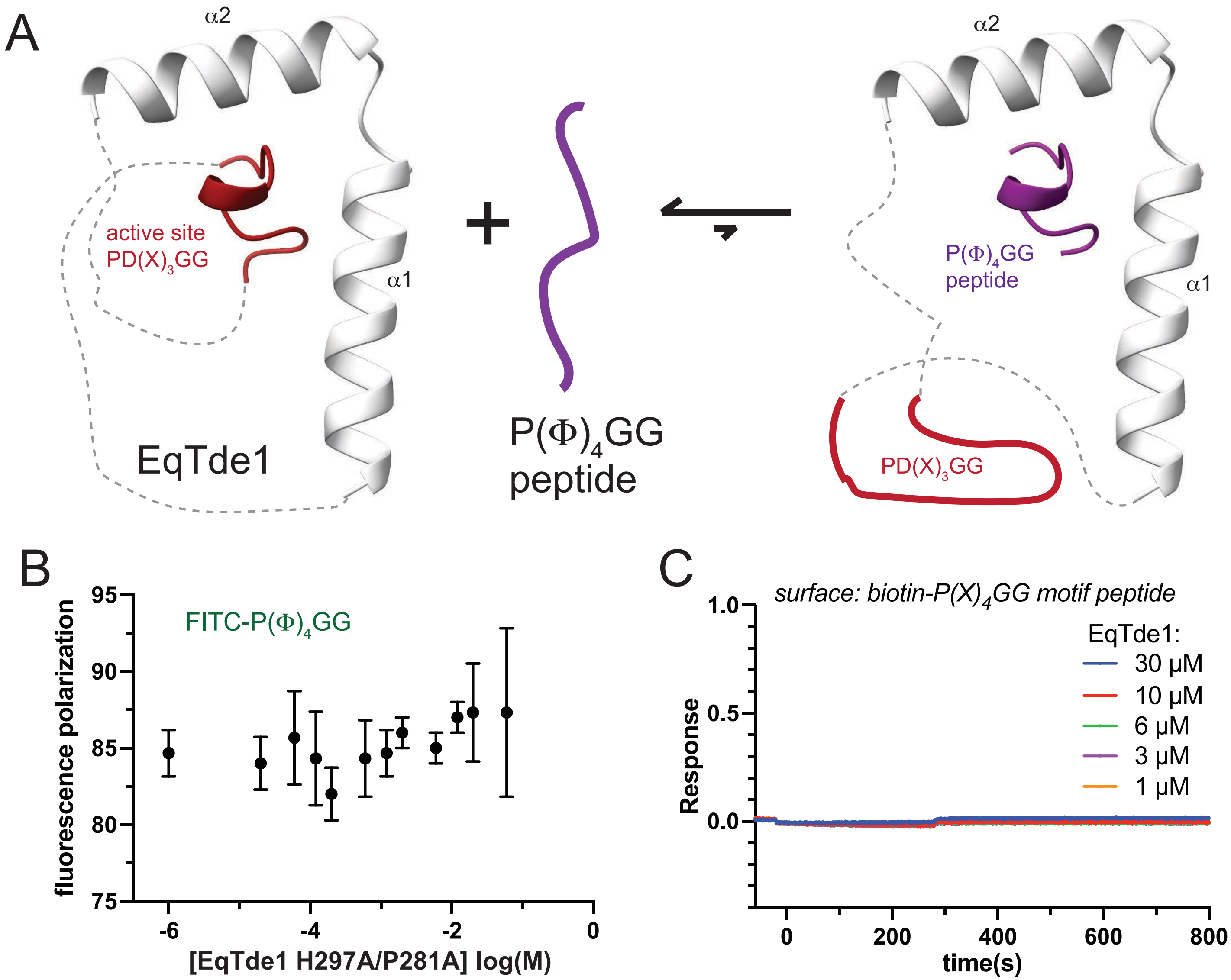

Figure S7. *Excess P(Φ)4GG motif peptide is required to competitively bind EqTde1.* (A) Because the endogenous PD$(X)_3$GG motif is covalently attached to EqTde, its positioning in the active site is thermodynamically favorable. High concentrations of P$(\Phi)_4$GG peptide are required to competitively displace this motif. (B) Fluorescence polarization with 10 nM FITC-P$(\Phi)_4$GG peptide and excess EqTde1 did not detect significant binding. (C) Similarly, excess EqTde1 analyte showed no significant binding to immobilized biotin-P$(\Phi)_4$GG peptide by BLI.

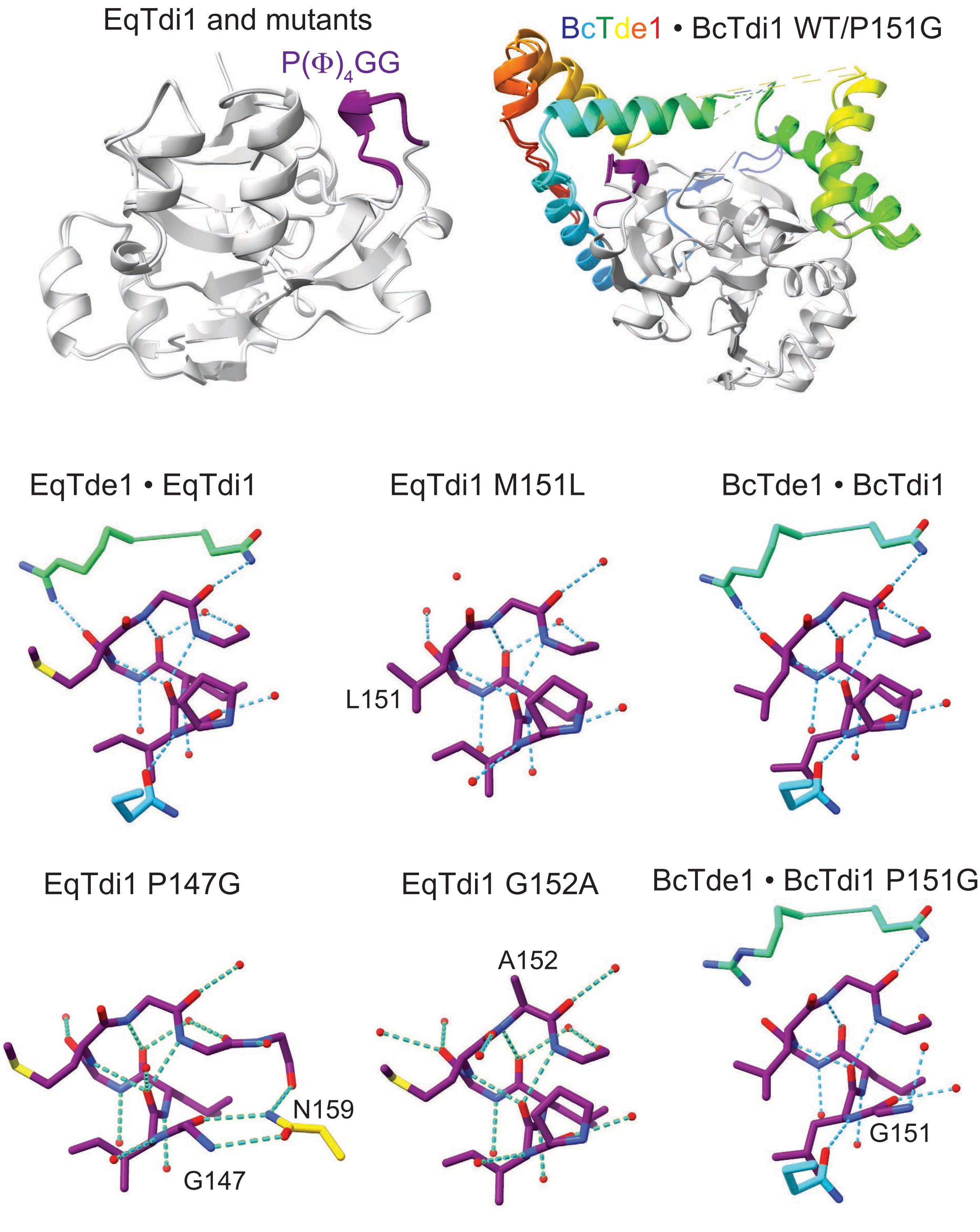

Figure S8. *Crystal structures of EqTdi1 and BcTdi1 P(Φ)$_4$GG motif mutants.* EqTdi1 mutants P147G, M151L, and G152A were purified in complex with EqTde1, but crystallized alone. Their overall fold is essentially identical to wild-type EqTdi1 in complex with EqTde1. Minimal H-bond network differences in the P(F)$_4$GG motif were observed. Specifically, the mutant glycine in P147G formed new H-bonds with N159 and rotated the psi angle of G153 to also interact with N159. BcTdi1 P151G was crystallized in complex with BcTde1, and was essentially identical to the wild type complex.
