# Supplementary material for: Secreted nuclease effector neutralization by active site mimicry in Bacillota
Source: mBio. 2026 Aug 17;17(9):e01516-26. doi: 10.1128/mbio.01516-26 (PMC13556189; doi:10.1128/mbio.01516-26)
Supplement: Supplementary file 2 [file mbio.01516-26-s0002.pdf]

Supplemental methods

Metagenome assembled genome bioinformatics

Tde and Tdi homologs were identified in ~200,000 metagenome assembled genomes (MAGs) from the Mgnify database (1) using BLAST and HMMer (2, 3). Taxonomy of the searched MAGs was visualized using R package Metacoder (4). Genes for *tde* were identified in 1010 MAGs, with the vast majority encoding adjacent *tdi*. Enrichment of *tde* and *tdi* was assessed with Z-score statistics comparing *tde/tdi*+ genomes within a taxonomic group compared to the detection rate in all MAGs. TXSScan (5) was applied to all *tde*-positive MAGs and *tde*-negative controls matched for genus to identify candidate secretion systems. Family-level secretion system enrichment was assessed with Chi-square statistics. Protein sequence cluster analysis on the same set of *tde*-positive MAGs and genus-matched controls was conducted with mmseq2 (6) on the predicted proteomes.

Escherichia coli toxicity

Escherichia coli BL21 cells were transformed with EqTde1 encoded on a “no-leak” arabinose-inducible promoter plasmid, pAra, and EqTdi1 maltose binding-protein (MBP) fusions encoded on an IPTG-inducible promoter plasmid, pET28a. Empty pAra and pET28a with MBP alone were used as controls. Transformed *E. coli* were grown overnight in LB medium supplemented with ampicillin and kanamycin, at 37 °C with shaking. Overnight cultures were diluted 1:100 in fresh LB with antibiotics and grown to early log phase. Protein expression was induced with 500 μM IPTG and 0.2% (w/v) L-arabinose for 3 hours. Serial dilutions were plated on LB agar containing appropriate antibiotics, and CFU were enumerated after overnight incubation. All experiments were performed at a minimum of biological triplicates, and statistical significance was determined using unpaired two-tailed t-tests.

Protein and peptide purification

All recombinant proteins used in this study were expressed from plasmids in BL21 *E. coli*, with or without pLysS. Cells were lysed with sonication in the presence of lysozyme and DNase I, and the soluble fraction was isolated by centrifugation. All proteins were isolated by hexahistidine tagging (tag on Tde in the Tde·Tdi complexes) and nickel nitrilotriacetic acid (NTA) affinity chromatography in buffers containing 30 mM HEPES pH 7.5, 300 mM NaCl, and variable concentrations of imidazole on an FPLC (Cytiva AKTA pure). The NTA eluates were further purified using size exclusion chromatography (Superdex 200) in 30 mM HEPES pH 7.5, 200 mM NaCl, and 1 mM dithiothreitol, concentrated, and snap frozen. EqTdi1 immunity only proteins were expressed and purified, fused to the C-terminus of hexahistidine-tagged maltose binding protein to improve solubility. Biotinylated MBP-EqTdi was produced using a C-terminal Avi-tag (pMal-T-Avi-His/BirA, a gift from Tonia Rex, Addgene plasmid #102962), co-expression of BirA from the same plasmid, and addition of biotin to the large-scale culture. Active purified EqTde1-hexahistidine was obtained by co-expression with EqTdi1 to avoid toxicity, denaturation in 6M guanidine, and on-column refolding using a linear gradient to gradually reduce the guanidine concentration to zero. The refolded protein was eluted from NTA and further purified with size exclusion chromatography to separate properly folded EqTde from misfolded and aggregated protein. For NMR experiments, ^{15}N-labeled EqTde(H279A/P281A) was produced by induction of expression for 3 hours at 37ºC in BL21 *E. coli* in M9 minimal media using $^{15}NH_4Cl$ (1 g/L) as the sole nitrogen source. Peptides were synthesized by and purchased from ABclonal Technology. The 14-residue peptides had sequence GYV**PILGMGG**MEKV, encompassing the $P(\Phi)_4GG$ motif and several upstream and downstream residues from EqTdi1. Two versions were created with either an N-terminal biotin moiety or an N-terminal FITC fluorophore with a 6-aminohexanoic acid linker.

Crystallography

All Tde·Tdi complexes were crystallized using hanging drop diffusion at 20 ºC with protein concentrations of 5-10 mg/mL. Crystallization screening was performed at the Hauptman-Woodward Research Institute National Crystallization Center (7). Optimization was performed in-house with manual drop diffusion plates. Crystallization solutions were: BcTde1·BcTdi1 – 18% w/v polyethylene glycol (PEG) 4000, 100 mM potassium thiocyanate, 100 mM citrate pH 4.5; BtTde1·BtTdi1 – 20% w/v PEG 3350, 200 mM sodium citrate; EqTde1·EqTdi1 – 25% w/v PEG 3350, 5% 1-n-butyl-3-methylimidazolium n-octyl sulfate, 90 mM Bis-Tris propane pH 8.8; EqTde1·EqTdi1(P147G) – 20% w/v PEG 20,000, 100 mM magnesium nitrate, 100 mM Bis-Tris propane pH 7.0; EqTde1·EqTdi1(G152A) – 22% w/v PEG 20,000, 50 mM sodium molybdate, 100 mM Bis-Tris pH 7.0 ; EqTde1·EqTdi1(M151L) – 22% w/v PEG 20,000, 100 mM potassium thiocyanate, 100 mM CAPS pH 10.0; BcTde1·BcTdi1(P151G) – 38% v/v PEG 400, 100 mM KCl, 100 mM sodium citrate pH 4.2; BcTde1·BcTdi1(G156R) – 20% w/v PEG 3350, 150 mM ammonium sulfate, 100 mM Bis-Tris pH 5.5; BcTde1·BcTdi1(G157R) – 20% w/v PEG 3350, 200 mM malonate pH 5.0.

Crystals were cryopreserved in crystallization solution supplemented with one of three cryoprotectants: 25% saturated sucrose, 25% glycerol, of 25% ethylene glycol. Crystals were flash frozen in liquid nitrogen for diffraction data collection at 77 K using beamline 4.2.2 at the Advanced Light Source, Berkeley National Lab. Diffraction data were scaled and merged using XDS, AIMLESS, and POINTLESS (8, 9). Resolution cutoff was selected based on an $I/\sigma I > 1.5$. All structures were solved using molecular replacement in Phenix (Phaser) and an AlphaFold 3 prediction model for the respective Tdi (10, 11). Models for Tde were built with a combination of Phenix Autobuild and manual revisions in Coot. (12, 13). Refinement was carried out using Phenix Refine (14). Refined structures will be deposited in the protein data bank at the time of publication.

Nuclear magnetic resonance (NMR) spectroscopy

1D ^{1}H and 2D ^{15}N/^{1}H HSQC NMR spectra were acquired on a Bruker Avance Neo 600 MHz NMR spectrometer equipped with a QCI cryoprobe at 25°C by using 100 μM uniformly ^{15}N-labeled EqTde1$^{H279A/P281A}$ alone or in complex with unlabeled MBP-EqTdi1 at molar ratios of 1:0.5, 1:1, and 1:1.5 (^{15}N-EqTde1$^{H279A/P281A}$: MBP-EqTdi1) in a NMR buffer containing 50 mM sodium phosphate (pH 5.5) and 50 mM NaCl. All NMR samples contain 90% H_2O / 10% D_2O in NMR buffer. ^{15}N-heteronuclear NOE experiments were also acquired on the ^{15}N-EqTde1$^{H279A/P281A}$ / MBP-EqTdi1 complex at 1:1.5 molar ratio (^{15}N-EqTde$^{H279A/P281A}$: MBP-EqTdi1) as previously described (15). The ^{1}H chemical shifts were referenced to 2,2-dimethyl-2-silapentane-5-sulfonate (DSS). The collected data were processed using NMRPipe (16) and analyzed using NMRView (17).

Structure and sequence bioinformatics

The amino acid sequences of 231 Tde·Tdi pairs identified in MAGs were trimmed to exclude fused domains and aligned using Clustal omega (18). Tde and Tdi phylogenetic trees were constructed and phylogenetic distances calculated using maximum-likelihood methods in MEGA (19). Residue positions corresponding to Tde·Tdi contacts in the crystal structures were isolated and sequence logos generated using WebLogo (20). AlphaFold 3 (10) models of 231 Tde·Tdi pairs identified in MAGs were predicted. All pairwise combinations were compared using Foldseek (21) and the resulting TM-scores. Generalized linear models were constructed in R (glm) with TM-score (log transformed) as the dependent variable, and taxonomic distance, Tdi phylogenetic distance, and Tde phylogenetic distance as the independent variables.

Limited trypsin proteolysis

Limited proteolysis was performed on ice with the protein(s) of interest at 4 ºC in buffer containing 50 mM HEPES pH 7.5, 1 mM EDTA, 10 mM $MgCl_2$, and 5 mM DTT. Digestion was

initiated by adding bovine pancreas trypsin (100 ng/mL) and halted at 2 hours with SDS loading buffer. Protein products were separated by SDS-PAGE and visualized with Coomassie blue staining. All experiments were performed in at least biological triplicate.

Differential scanning fluorimetry

Recombinant EqTde1·EqTdi1 complexes at 5 μM in 30 mM HEPES pH 7.5, 200 mM NaCl, and 1 mM dithiothreitol were mixed with SYPRO orange dye at 3X concentration in 96-well qPCR plates. The temperature was increased at 0.05 ºC/s with fluorescence measurements every 0.5 ºC in a qPCR thermocycler (QuantStudio 3). Melting temperatures were derived from the inflection point using first derivative curves. All experiments were performed in at least biological triplicate. Melting temperatures were compared using unpaired Student's t-tests.

Biolayer interferometry

Binding experiments between EqTde1 and EqTdi1 were conducted using an Octet Red96 instrument (Sartorius). Immunity proteins were fused to maltose-binding protein (MBP) and biotinylated. EqTdi1 at 10 mg/mL was immobilized onto streptavidin biosensors. EqTde1 H279A/P281A at increasing concentrations in Octet kinetics buffer (Sartorius) was allowed to associate (300 s) and dissociate (600 s) to obtain equilibrium binding dose-response curves. Dissociation constants were calculated using one-site binding curve fitting of the equilibrium binding data in Prism (GraphPad), following subtraction of non-specific binding to an irrelevant control surface (biotin only). For EqTde1 H279A/P281A and peptide binding experiments, one partner was immobilized in NTA biosensors (EqTde1) or streptavidin biosensors (biotin-P(Φ)4GG peptide). The other component was used as an analyte in a buffer containing 30 mM HEPES (pH 7.5), 200 mM NaCl, and 0.05% v/v Tween-20. Non-specific binding controls were biotin-only (for streptavidin) and no protein loading for NTA. Association and dissociation

intervals were 300 and 600 s, respectively. All presented data represent at least biological duplicate experiments.

Fluorescence polarization

Fluorescence polarization was measured using a BioTek Synergy H1 plate reader. FITC-labeled P(Φ)$_4$GG peptide at 100 nM was mixed with increasing concentrations of EqTde1 H279A/P281A or DMSO vehicle control in buffer containing 30 mM HEPES pH 7.5, 200 mM NaCl, and 0.05% v/v Tween-20. All experiments were performed in technical triplicate, and the presented data are representative of two independent experiments.

Nuclease activity

Nuclease activity was detected using a quenched fluorophore oligonucleotide substrate (DNaseAlert, Integrated DNA Technologies) and a TECAN Infinite M200 plate reader. Fluorescent oligonucleotide substrate (1μM) was incubated at 37°C with 1x DNase Reaction Buffer (IDT) containing either recombinant purified MBP-EqTdi1 (5 μM), EDTA (1 mM), or nuclease-free water. For peptide inhibition experiments, the P(Φ)$_4$GG motif peptide with biotin was incubated with substrate oligonucleotide at increasing concentrations. Reactions were initiated by the addition of refolded EqTde1 (0.5 μM). Cleaved fluorogenic DNA was measured over 1 hour at 556 nm emission. Bovine pancreas-derived DNase I was used as a positive control. Data presented are at least technical triplicate and representative of at least 3 independent experiments.

Secretomes

Secretomes were measured from log-phase *B. thuringiensis* ATCC 10792 cultures in 250 mL Luria broth. Cells were removed by centrifugation, and proteins were precipitated from the

conditioned media using 8% v/v trichloroacetic acid at 4 ºC overnight. The precipitant was isolated by centrifugation, resuspended in deionized water, and reprecipitated in 85% pre-chilled acetone at -20 ºC for two hours. The precipitant was isolated by centrifugation (18,000 x g for 30 minutes). The acetone supernatant was discarded and the pellet was dried under vacuum, then resuspended in 8M urea, 75 mM NaCl, and 50 mM Tris pH 8.2. Total protein recovery was quantified with bicinchoninic acid assay. Proteins were reduced with 5 mM DTT and alkylated with 14 mM iodoacetamide. The urea concentration was reduced to 1.6 M by addition of 25 mM Tris pH 8.2. Trypsin proteolysis was initiated by adding activated trypsin at a 1:200 ratio to total protein with 1 mM calcium chloride, incubated overnight at 37 ºC. Digestion was halted by adding 0.4% v/v trifluoroacetic acid. The resulting peptide solution was cleared with centrifugation and passed through a C18 column (Nest Group BioPureSPN 300-350 μg capacity), with elution in 80% acetonitrile and 25 mM formic acid. Peptides were dried at 37 ºC and stored at -20 ºC. Peptides were identified and measured with label-free quantitation on a Thermo Lumos tribrid mass spectrometer, using orbitrap ESI MS/MS and data-driven data collection protocols. For nano-liquid chromatography, peptides were resuspended in at 1 μg/μL in water, and a 1 μg injection was separated over a C18 column with a linear gradient of acetonitrile over 1 hour. Peptides were identified and quantified using Maxquant (22).

Mass spectrometry protein identification

Limited trypsin proteolysis fragments were identified by staining protein electrophoresis gels with MS safe blue stain (Simplyblue Safestain). Bands were excised, in-gel digested with trypsin, and passed through a C18 column. Peptides were dried at 37 ºC and stored at -20 ºC. Peptides were identified on a Thermo Lumos tribrid mass spectrometer and mapped to the EqTde1 and MBP-EqTdi1 sequences.

HiBiT expression quantification

EqTdi1 and mutants were fused with a C-terminal HiBiT peptide tag (VSGWRLFKKIS) and delivered to Bt HD73 in an ICE, as described in the main text methods. Bt HD73 strains were grown in liquid media with or without 1 mM IPTG to induce expression. Bacteria were lysed with sonication and lysozyme (0.5 mg/mL), and total protein was normalized using a Bradford assay. HiBiT tags were detected by complementation of nanoluciferase in the presence of furimazine and ATP (Nano-Glo HiBiT, Promega). Bioluminescence was quantified in white 96-well plates using a BioTek Synergy reader.

References

1. Richardson L, Allen B, Baldi G, Beracochea M, Bileschi ML, Burdett T, Burgin J, Caballero-Perez J, Cochrane G, Colwell LJ, Curtis T, Escobar-Zepeda A, Gurbich TA, Kale V, Korobeynikov A, Raj S, Rogers AB, Sakharova E, Sanchez S, Wilkinson DJ, Finn RD. 2023. MGnify: the microbiome sequence data analysis resource in 2023. Nucleic Acids Res 51:D753–D759.
2. Bosch DE, Abbasian R, Parajuli B, Peterson SB, Mougous JD. 2023. Structural disruption of Ntox15 nuclease effector domains by immunity proteins protects against type VI secretion system intoxication in Bacteroidales. mBio 14:e0103923.
3. Potter SC, Luciani A, Eddy SR, Park Y, Lopez R, Finn RD. 2018. HMMER web server: 2018 update. Nucleic Acids Res 46:W200–W204.
4. Foster ZS, Sharpton TJ, Grunwald NJ. 2017. Metacoder: An R package for visualization and manipulation of community taxonomic diversity data. PLoS Comput Biol 13:e1005404.
5. Abby SS, Denise R, Rocha EPC. 2024. Identification of Protein Secretion Systems in Bacterial Genomes Using MacSyFinder Version 2. Methods Mol Biol 2715:1–25.
6. Steinegger M, Söding J. 2017. MMseqs2 enables sensitive protein sequence searching for the analysis of massive data sets. Nat Biotechnol 35:1026–1028.
7. Budziszewski GR, Snell ME, Wright TR, Lynch ML, Bowman SEJ. 2023. High-Throughput Screening to Obtain Crystal Hits for Protein Crystallography. J Vis Exp doi:10.3791/65211.
8. Kabsch W. 2010. Xds. Acta Crystallogr D Biol Crystallogr 66:125–32.
9. Agirre J, Atanasova M, Bagdonas H, Ballard CB, Basle A, Beilsten-Edmands J, Borges RJ, Brown DG, Burgos-Marmol JJ, Berrisford JM, Bond PS, Caballero I, Catapano L, Chojnowski G, Cook AG, Cowtan KD, Croll TI, Debreczeni JE, Devenish NE, Dodson EJ, Drevon TR, Emsley P, Evans G, Evans PR, Fando M, Foadi J, Fuentes-Montero L, Garman EF, Gerstel M, Gildea RJ, Hatti K, Hekkelman ML, Heuser P, Hoh SW, Hough MA, Jenkins HT, Jimenez E, Joosten RP, Keegan RM, Keep N, Krissinel EB, Kolenko P, Kovalevskiy O, Lamzin VS, Lawson DM, Lebedev AA, Leslie AGW, Lohkamp B, Long F, Maly M, et al. 2023. The CCP4 suite: integrative software for macromolecular crystallography. Acta Crystallogr D Struct Biol 79:449–461.
10. Abramson J, Adler J, Dunger J, Evans R, Green T, Pritzel A, Ronneberger O, Willmore L, Ballard AJ, Bambrick J, Bodenstein SW, Evans DA, Hung CC, O'Neill M, Reiman D, Tunyasuvunakool K, Wu Z, Zemgulyte A, Arvaniti E, Beattie C, Bertolli O, Bridgland A, Cherepanov A, Congreve M, Cowen-Rivers AI, Cowie A, Figurnov M, Fuchs FB, Gladman H, Jain R, Khan YA, Low CMR, Perlin K, Potapenko A, Savy P, Singh S, Stecula A, Thillaisundaram A, Tong C, Yakneen S, Zhong ED, Zielinski M, Zidek A, Bapst V, Kohli P, Jaderberg M, Hassabis D, Jumper JM. 2024. Accurate structure prediction of biomolecular interactions with AlphaFold 3. Nature 630:493–500.
11. Liebschner D, Afonine PV, Baker ML, Bunkoczi G, Chen VB, Croll TI, Hintze B, Hung LW, Jain S, McCoy AJ, Moriarty NW, Oeffner RD, Poon BK, Prisant MG, Read RJ, Richardson JS, Richardson DC, Sammito MD, Sobolev OV, Stockwell DH, Terwilliger TC, Urzhumtsev AG, Videau LL, Williams CJ, Adams PD. 2019. Macromolecular structure determination using X-rays, neutrons and electrons: recent developments in Phenix. Acta Crystallogr D Struct Biol 75:861–877.
12. Casañal A, Lohkamp B, Emsley P. 2020. Current developments in Coot for macromolecular model building of Electron Cryo-microscopy and Crystallographic Data. Protein Sci 29:1069–1078.

13. Terwilliger TC, Grosse-Kunstleve RW, Afonine PV, Moriarty NW, Zwart PH, Hung LW, Read RJ, Adams PD. 2008. Iterative model building, structure refinement and density modification with the PHENIX AutoBuild wizard. Acta Crystallogr D Biol Crystallogr 64:61–9.
14. Afonine PV, Grosse-Kunstleve RW, Echols N, Headd JJ, Moriarty NW, Mustyakimov M, Terwilliger TC, Urzhumtsev A, Zwart PH, Adams PD. 2012. Towards automated crystallographic structure refinement with phenix.refine. Acta Crystallogr D Biol Crystallogr 68:352–67.
15. Yu L, Zhu CX, Tse-Dinh YC, Fesik SW. 1996. Backbone dynamics of the C-terminal domain of Escherichia coli topoisomerase I in the absence and presence of single-stranded DNA. Biochemistry 35:9661–6.
16. Delaglio F, Grzesiek S, Vuister GW, Zhu G, Pfeifer J, Bax A. 1995. NMRPipe: a multidimensional spectral processing system based on UNIX pipes. J Biomol NMR 6:277–93.
17. Johnson BA, Blevins RA. 1994. NMR View: A computer program for the visualization and analysis of NMR data. J Biomol NMR 4:603–14.
18. Sievers F, Higgins DG. 2014. Clustal omega. Curr Protoc Bioinformatics 48:3.13.1–16.
19. Stecher G, Suleski M, Tao Q, Tamura K, Kumar S. 2025. MEGA 12.1: Cross-Platform Release for macOS and Linux Operating Systems. J Mol Evol doi:10.1007/s00239-025-10287-z.
20. Crooks GE, Hon G, Chandonia JM, Brenner SE. 2004. WebLogo: a sequence logo generator. Genome Res 14:1188–90.
21. van Kempen M, Kim SS, Tumescheit C, Mirdita M, Lee J, Gilchrist CLM, Soding J, Steinegger M. 2024. Fast and accurate protein structure search with Foldseek. Nat Biotechnol 42:243–246.
22. Prianichnikov N, Koch H, Koch S, Lubeck M, Heilig R, Brehmer S, Fischer R, Cox J. 2020. MaxQuant Software for Ion Mobility Enhanced Shotgun Proteomics. Mol Cell Proteomics 19:1058–1069.
